# Design of Functional Food Containing Encapsulated Bioactive Compounds Stabilized in a Psyllium–Potato Starch System

**DOI:** 10.3390/ijms27135685

**Published:** 2026-06-24

**Authors:** Magdalena Krystyjan, Mariola Kmita, Gohar Khachatryan, Karen Khachatryan, Anna Lenart-Boroń, Robert Socha, Anna Areczuk, Joanna Sobolewska-Zielińska

**Affiliations:** 1 Department of Carbohydrates Technology and Cereal Processing, Faculty of Food Technology, University of Agriculture in Krakow, Mickiewicza Ave. 21, 31-120 Krakow, Poland; anna.areczuk@urk.edu.pl; 2 Food Technologists Scientific Circle, Carbohydrate Technology Section, University of Agriculture in Krakow, Mickiewicza Ave. 21, 31-120 Krakow, Poland; mariola.kmita@student.urk.edu.pl; 3 Department of Food Analysis and Quality Assessment, Faculty of Food Technology, University of Agriculture in Krakow, Mickiewicza Ave. 21, 31-120 Krakow, Poland; gohar.khachatryan@urk.edu.pl (G.K.); robert.socha@urk.edu.pl (R.S.); joanna.sobolewska-zielinska@urk.edu.pl (J.S.-Z.); 4 Laboratory of Nanotechnology and Nanomaterials, Faculty of Food Technology, University of Agriculture in Krakow, Mickiewicza Ave. 21, 31-120 Krakow, Poland; karen.khachatryan@urk.edu.pl; 5 Department of Microbiology and Biomonitoring, Faculty of Agriculture and Economics, University of Agriculture in Krakow, 30-059 Krakow, Poland; anna.lenart-boron@urk.edu.pl; 6 Centre for Innovation and Research on Prohealthy and Safe Food, University of Agriculture in Krakow, Mickiewicza Ave. 21, 31-120 Krakow, Poland

**Keywords:** encapsulation, potato starch, psyllium, bioactive compounds, plant extracts, novel foods, functional foods

## Abstract

This research focused on the formulation of a health-oriented, clean-label food product fortified with encapsulated bioactive compounds from *Sambucus nigra*, *Aronia melanocarpa*, and *Echinacea purpurea*. To evaluate the protection of these sensitive compounds during production and storage, a comprehensive characterization was performed. This included basic physicochemical analyses, phenolic profiling, antioxidant activity tests, as well as rheological and textural measurements. Furthermore, sensory analysis, consumer evaluation, and microbiological stability during storage were assessed. Results from Scanning Electron Microscopy (SEM) and Fourier-Transform Infrared Spectroscopy (FTIR) analyses confirmed the structural integrity of the capsules post-processing. Additionally, the application of a starch–psyllium carrier ensured that the textural and rheological properties remained fully comparable to the control sample, preventing undesirable matrix alterations. Specifically, product hardness (1.17–1.23 N) and adhesiveness (8.17–8.94 N·s) were maintained at stable levels, while color alterations were minor and likely noticeable only to trained observers (ΔE* < 3.2). Microbiological evaluation demonstrated that the application of different formulated products effectively inhibited the growth of Gram-positive and Gram-negative bacterial strains, with inhibition rates increasing from 3.4 to 39.7%. Collectively, the experimental data demonstrate that encapsulation is a highly effective strategy for fortifying fruit-based systems with sensitive extracts, successfully maximizing bioactivity retention while maintaining high product quality and sensory appeal.

## 1. Introduction

Current food market trends are driven by growing consumer awareness. As healthy lifestyles continue to gain traction, consumers increasingly seek products with high nutritional and health-promoting value. A clear shift away from highly processed foods toward ingredients that support overall well-being is driving the rapid expansion of the functional food sector. In fact, its global market value is projected to reach $91 billion in the near future [1]. The modern diet frequently relies on highly processed foods that are calorie-dense yet deficient in essential micronutrients. Advanced technological processing and refining strip the final products of most of their natural bioactive compounds, drastically diminishing their actual health benefits. This type of diet directly contributes to vitamin and mineral deficiencies and fosters the development of chronic, diet-related diseases, such as colorectal cancer and type 2 diabetes [2,3]. The functional food sector has emerged as a response to these negative trends, offering products formulated to deliver significantly higher concentrations of key active substances than their traditional or processed counterparts. Regular consumption of foods fortified with vitamins, antioxidants, prebiotics, or fiber not only bridges daily nutritional gaps but also actively maintains the body’s physiological balance. Furthermore, these benefits extend beyond physical health to positively impact the nervous system and overall mental well-being, establishing functional foods as a cornerstone of modern preventive healthcare [4,5,6]. However, enriching food with health-promoting substances presents a challenge that requires advanced technological solutions. Many bioactive compounds—such as vitamins, unsaturated fatty acids, and live probiotic cultures—are highly unstable and prone to oxidation [7]. Without appropriate protective measures, these ingredients could degrade during manufacturing, storage, or even upon contact with gastric acid following consumption. Consequently, the use of modern delivery systems—such as micro- and nanoencapsulation or nanoemulsions—has become crucial. These technologies effectively isolate active substances from harsh external environments. Not only do they extend product shelf life, but more importantly, they enhance the bioavailability of the ingredients, ensuring optimal absorption by the body. As a result, essential micronutrients can be successfully delivered through functional foods, exerting a profoundly positive impact on overall health [8,9,10].

Plant-based immunostimulants bolster the immune system by stimulating antibody production and activating the body’s natural defense mechanisms, thereby providing protection against viral, bacterial, and fungal infections [11]. Regular consumption of these compounds serves as a crucial preventive measure, particularly during periods of heightened susceptibility to illness. The primary bioactive compounds found in plants such as elderberry, chokeberry, and echinacea are polyphenols, encompassing phenolic acids, flavonoids, and anthocyanins. These compounds exhibit potent antioxidant activity, shielding cells from oxidative stress. Furthermore, their anti-inflammatory, antiviral, and antibacterial properties synergistically support optimal immune function. Beyond immunity, these phytonutrients positively impact the cardiovascular system and metabolism while offering neuroprotective benefits. Specifically, anthocyanins strengthen blood vessels, whereas phenolic acids can protect the skin from UV-induced damage. Additionally, oat-derived β-glucans play a significant role in maintaining healthy blood cholesterol levels and attenuating postprandial blood glucose spikes [12,13,14,15,16].

Many bioactive compounds exhibit limited stability under standard food processing conditions. Exposure to factors such as oxygen, elevated temperatures, light, pH fluctuations, enzymatic activity, and interactions with proteins or polysaccharides can trigger the degradation, oxidation, or structural alteration of these sensitive molecules. Consequently, the direct fortification of food products with extracts or oils frequently results in a loss of biological activity, undesirable quality changes—such as color degradation or the development of off-flavors—and reduced product stability throughout its shelf life [17,18,19].

One of the most viable and frequently applied solutions is the encapsulation of bioactive compounds. This method mitigates adverse phenomena by physically protecting the active substances within a carrier matrix, thereby reducing their exposure to the external environment and minimizing degradation reactions [20,21]. Beyond its protective function, encapsulation facilitates the technological integration of bioactive ingredients into complex food matrices. Entrapping active compounds within micro- or nanocarrier structures can diminish the intensity of bitterness and astringency characteristic of many plant extracts, while simultaneously improving their dispersion in high-water-content systems. This is of particular importance in products such as fruit purée and mousses, which require a homogeneous, smooth texture and long-term shelf stability [22,23,24,25,26].

Another crucial aspect of encapsulation is the ability to tailor the release profile of bioactive compounds. A properly designed carrier can ensure controlled release during both storage and digestion, potentially enhancing bioactivity by improving overall bioavailability. This is especially significant for compounds susceptible to degradation in acidic environments or in the presence of enzymes, as well as for poorly soluble ingredients, where the encapsulated form can facilitate transport and targeted release within the gastrointestinal tract [27,28,29].

Despite the variety of available encapsulation techniques, selecting the appropriate method depends on the nature of the active substance (hydrophilic vs. lipophilic), the targeted particle size, processing constraints, and the final application in the product [30,31]. For fruit purées and mousses, highly desirable carriers are those that provide high encapsulation efficiency, resistance to oxidation and pH fluctuations, compatibility with high water activity, and minimal impact on quality attributes such as color, flavor, and texture. Therefore, designing an encapsulation system must bridge functional requirements with technological and sensory demands, ensuring that fortification enhances the product’s health-promoting value without compromising consumer acceptability [32,33].

Polysaccharides are natural food components widely utilized due to their broad spectrum of functional properties and high abundance. Potato starch exhibits excellent compatibility with food systems, owing to its network-forming capabilities, complexation potential, and structure-forming properties [34,35]. Consequently, it has found numerous applications throughout the food industry. However, native starches possess certain limitations that can restrict their processing and application. Minimizing these drawbacks through synergistic interactions with other polymers enables their controlled and targeted utilization [36,37,38].

Psyllium comprises polysaccharides with specific, well-documented properties. Psyllium mucilage exhibits robust hydrogel-forming and viscoelastic characteristics, significantly influencing the texture and stability of the resulting gels. It is classified as a soluble fraction of dietary fiber and demonstrates substantial prebiotic potential. The synergistic combination of these two polymers—starch and psyllium—can potentially enhances’ capsule stability and their dispersion within a purée, mitigate the loss of bioactive substances, and favorably modify the rheological properties of the systems [39]. Our previous studies have confirmed the occurrence of synergy between these two polysaccharides. The addition of mucilage reduced the decomposition of the system, which made the starch/psyllium films more thermally stable [40]. Furthermore, we have verified the feasibility of encapsulating bioactive compounds using this polysaccharide matrix as a carrier, along with its stability following the lyophilization process [39]. Through these applied techniques, biocomposites with a stable starch/psyllium matrix were developed, which not only served as an efficient carrier for bioactive compounds but also provided effective protection against their degradation.

Numerous studies have documented the fortification of food with extracts, oils, and various bioactive compounds, applied either directly or through encapsulation within starches and other biopolymers. However, the synergistic properties of binary starch/psyllium systems have not yet been utilized for the encapsulation of bioactive compounds. In light of the above, the primary aim of this study is to develop a clean-label functional food: an oat-fortified fruit purée enriched with encapsulated grape seed oil and bioactive substances derived from elderberry, aronia, and echinacea. This study evaluates the encapsulation efficiency of a binary matrix composed of potato starch and psyllium. We hypothesize that this collaborative biopolymer system will provide superior protection against the oxidative and thermal degradation of sensitive compounds, thereby extending their stability. Furthermore, the incorporation of the starch–psyllium carrier is expected to significantly improve the textural and rheological integrity of the food matrix without adversely affecting its sensory profile, successfully bridging the gap between high nutritional value and consumer appeal.

## 2. Results and Discussion

### 2.1. Morphological Characterization of Enriched Fruit Products by SEM Analysis

**Figure 1:**
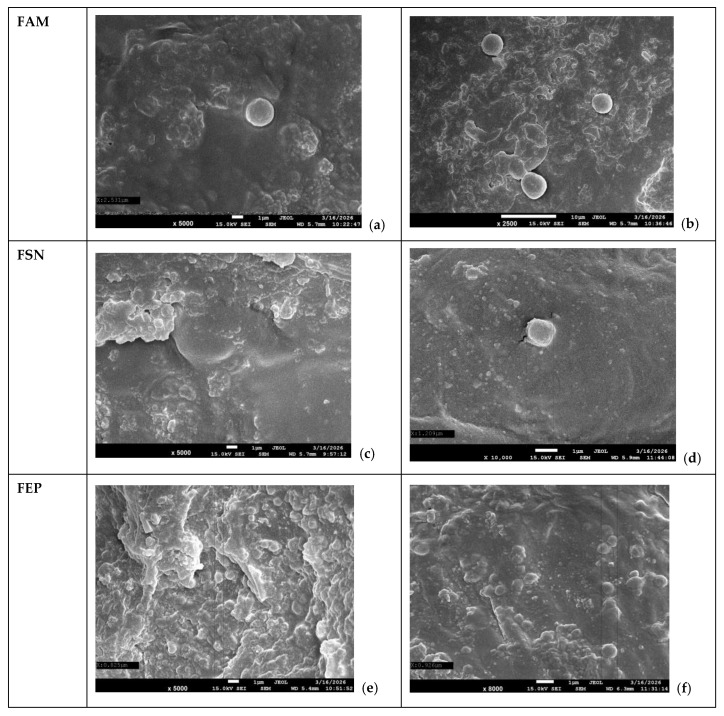
SEM micrographs of fruit purées with oat flakes enriched with polysaccharide-based biocomposites loaded with plant extracts: (**a**,**b**) FAM, enriched with *Aronia melanocarpa* biocomposites; (**c**,**d**) FSN, enriched with *Sambucus nigra* biocomposites; (**e**,**f**) FEP, enriched with *Echinacea purpurea* biocomposites.

SEM was used to assess the integrity of the polysaccharide-based nanocapsules after cooking and jar pasteurization. In the baseline study, the lyophilized starch/psyllium biocomposites formed a porous, sponge-like scaffold containing extract-loaded oil droplets of approximately 800–1500 nm [39]. After incorporation into the fruit purée with oat flakes, distinct, extract-dependent morphological rearrangements were observed (Figure 1).

FAM (fruit purée with oat flakes enriched with *Aronia melanocarpa* (chokeberry fruit) biocomposites) contained smooth, spherical microstructures embedded in the food matrix; their diameter had shifted from the nanoscale to several micrometers, confirming that the starch/psyllium carrier withstood blending and pasteurization. FSN (fruit purée enriched with *Sambucus nigra* (elderberry) biocomposites) showed spherical to slightly irregular, multifaceted structures with markedly rougher surfaces, tightly embedded in the surrounding fruit–oat network. FEP (fruit purée with oat flakes enriched with *Echinacea purpurea* (purple coneflower) biocomposites), by contrast, formed a dense, corrugated, continuous hydrogel-like network with aggregated nodular features in which individual capsules were no longer distinct.

These changes reflect two complementary mechanisms. First, the dry porous lyophilizates hydrate rapidly in the moisture-rich, low-pH purée; the high water-binding capacity of starch, and especially psyllium mucilage, expands the capsule boundaries into the micrometer range. Second, cooking and jar pasteurization break down the apple, strawberry and banana cell walls, releasing native pectin that acts as effective emulsifying and film-forming agents [41,42]. The anionic pectin chains migrate to the capsule surface, where hydrogen bonding with the starch/psyllium hydroxyl groups drives in situ self-assembly of a hybrid envelope around the oil droplets—a layer-by-layer coating that accounts for the increased thickness and surface roughness seen by SEM.

The contrast between FEP and FAM reflects extract chemistry. The EP extract is rich in caffeic acid derivatives [39]. The resulting abundance of polar phenolic hydroxyl groups, reflected in the highest polar component of surface free energy measured by FTIR, provides numerous hydrophilic sites that cross-link extensively with the released pectin, agglomerating the droplets into a continuous, reinforced network. FAM, in contrast, has a much lower polar component and a thermal plasticizing effect driven by its high content of low-molecular-weight sugars, so cross-linking was limited and the capsules expanded by hydration while retaining a smooth, intact spherical form.

The survival of these microstructures through pasteurization is significant for functional food design, since thermal processing typically causes emulsion collapse and rapid degradation of heat-sensitive anthocyanins and phenolics. Baseline DSC (Differential Scanning Calorimetry) data already showed that the starch/psyllium backbone shifts the exothermic degradation thresholds upwards via polyphenol–starch interactions [39]. The present results confirm that the fruit matrix actively reinforces the encapsulates. This multilayered hybrid architecture can mask herbal off-notes, protect pigments from oxidation during storage, and potentially enable controlled release of immunomodulatory compounds in the gastrointestinal tract.

### 2.2. Attenuated Total Reflectance Fourier-Transform Infrared Spectroscopy (ATR-FTIR)

**Figure 2:**
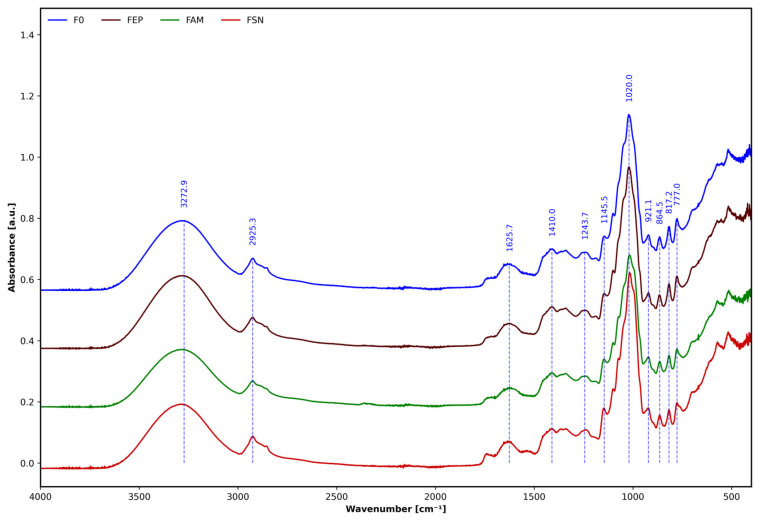
ATR-FTIR spectra of fruit sample variants: F0 (control), FSN (elderberry-enriched), FAM (chokeberry-enriched), and FEP (echinacea-enriched). Key absorption bands are labeled with their respective wavenumber positions (cm^−1^). Spectra are offset on the absorbance axis for clarity.

ATR-FTIR spectroscopy was employed to assess the chemical composition and structural integrity of the fruit puree variants—F0 (control sample - fruit purée with oat flakes, with the addition of freeze-dried potato starch gel), FSN (elderberry), FAM (chokeberry), and FEP (echinacea)—and to verify whether the incorporation of psyllium/starch-based biocomposites introduced any new covalent bonds or fundamentally altered the matrix chemistry after pasteurization. The spectra were recorded across the 4000–700 cm^−1^ range (Figure 2), and band assignments for all samples were based on the profile of the F0 reference spectrum, given the high degree of similarity observed across all variants.

All four spectra displayed the same characteristic absorption bands, confirming that the dominant infrared signal originated from the fruit/oat matrix rather than from the encapsulated plant extracts or the polysaccharide carrier itself. This is consistent with findings reported by Krystyjan et al. [40], who showed that starch–psyllium films exhibit a conserved polysaccharide fingerprint in FTIR spectra, and with our previous study demonstrating that psyllium/starch biocomposites retain the spectral signature of the carrier after lyophilization [39].

The broad absorption band centered at 3272.9 cm^−1^ is attributable to O–H stretching vibrations of hydroxyl groups and, to a lesser extent, N–H stretching from proteinaceous constituents of the oat fraction [43,44]. The broad profile of this band is characteristic of extensive intermolecular hydrogen bonding within the polysaccharide network, a feature commonly reported for starch- and psyllium-containing systems [40,45]. Hydrogen bonding plays a central role in the structural cohesion of the matrix; Krystyjan and Khachatryan (2017) highlighted that mucilage reinforcement of starch films is primarily mediated through such non-covalent O–H···O interactions [40]. The band at 2925.3 cm^−1^ corresponds to asymmetric C–H stretching of –CH_2_ and –CH_3_ groups, reflecting aliphatic carbon chains present in the fruit lipid fraction and grape seed oil entrapped within the capsules [44]. Its moderate intensity and relatively narrow profile suggest that the encapsulated oil did not disrupt the polysaccharide network, which is consistent with the rheological and textural data discussed in Section 2.6 and Section 2.7.

The band at 1625.7 cm^−1^ can be assigned to C = O stretching in the Amide I region, associated with β-sheet contributions from the oat protein fraction, and/or to C = O stretching of carbonyl groups of pectin present in the fruit matrix [43,46]. A related signal at 1410.0 cm^−1^ is attributed to symmetrical COO^−^ stretching and C–H bending vibrations, typical of carboxylate groups in pectins and organic acids [44,46]. These carboxylate groups play an important role in the gel-forming capacity of pectins under the acidic conditions (pH 4.08–4.11) encountered in the tested fruit products (Section 2.3). The band at 1243.7 cm^−1^ is characteristic of C–O–C ether stretching vibrations within the polysaccharide backbone, while the shoulder at 1145.5 cm^−1^ reflects C–O stretching within the glucopyranose ring of starch [45,47].

The most intense band in the fingerprint region, centered at 1020.0 cm^−1^, is characteristic of C–O–C and C–O stretching vibrations within the polysaccharide region and is a definitive marker of starch and psyllium mucilage [45,47]. This band is consistently reported as the dominant feature in FTIR spectra of potato starch and psyllium-based materials [3,39,40]. Its high relative intensity in all variants further confirms the structural dominance of the polysaccharide matrix, even after pasteurization and the incorporation of biocomposites. The band at 921.1 cm^−1^ is assigned to α-glycosidic linkage C–H deformation, a fingerprint feature of starch [47,48]. In potato starch, this band is particularly prominent and reflects the helical structure of amylose chains [48]. The cluster of bands at 864.5, 817.2, and 777.0 cm^−1^ is attributed to aromatic C–H out-of-plane deformation vibrations and carbonate-related vibrational modes [44,46].

Crucially, no new bands were observed in the spectra of FSN, FAM, or FEP that were absent from the control F0, and no significant band shifts were detected. This indicates that the incorporation of the psyllium/starch biocomposites into the sample did not induce new covalent interactions between the encapsulated substances and the fruit matrix. Instead, the stability of the system is governed by non-covalent forces—primarily hydrogen bonding, hydrophobic interactions, and electrostatic contacts—as previously documented for analogous starch-based encapsulation systems [39,40]. These findings are consistent with the results of Janik et al. [49], who reported an equivalent conservation of the polysaccharide spectral profile after functionalization of chitosan/alginate matrices with encapsulated oils, concluding that FTIR band concordance is a reliable indicator of matrix compatibility. Similarly, Krystyjan et al. [39] demonstrated by FTIR that the starch–psyllium matrix hosting *Sambucus nigra*, *Aronia melanocarpa*, and *Echinacea purpurea* extracts retained the characteristic polysaccharide bands after lyophilization, with differences limited to relative band intensities attributable to the phenolic content of each extract. The present results extend this observation to a pasteurized food matrix, confirming that the thermal treatment did not disrupt the structural integrity of the capsules—a conclusion corroborated by the SEM images discussed in Section 2.1, which revealed the persistence of intact spherical submicron structures within the dried purée variants.

The FTIR data, taken together with the polyphenol, rheological, and textural results (Section 2.4, Section 2.5, Section 2.6 and Section 2.7), support the conclusion that the starch–psyllium binary carrier functions as a chemically inert, physically protective shell that successfully isolates the bioactive substances from the surrounding food matrix throughout processing.

### 2.3. Basic Parameters of Products

Table 1 presents the basic physicochemical characteristics of the analyzed fruit purée with oat flakes, including water content, water activity (a_w_), pH, and total titratable acidity (TTA). Overall, the samples showed similar aw values (0.93), indicating comparable water availability across formulations. Small but statistically significant differences were observed in water content and acidity parameters. The FAM products exhibited the highest moisture level, whereas the remaining variants showed slightly lower values. The pH ranged narrowly from 4.08 to 4.11, with FSN showing the highest pH. In terms of acidity, FEP was characterized by the highest total titratable acidity, while FSN showed the lowest acidity.

The water activity (a_w_) of the fruit products obtained in this study was higher than that of the fruit products prepared in our previous research, which lacked the addition of oat flakes and utilized different ingredient proportions [26]. While the inclusion of oat flakes may have influenced the total water content, reducing it by 5 percentage points compared to our earlier findings, the observed increase in a_w_ and pH should be attributed to the variations in the formulation ratios.

It is also noteworthy that these parameters may be influenced by the specific apple cultivars used during production. Research conducted by Loncaric et al. [50] supports this assumption, showing that the pH of juices obtained from different apple varieties ranged from 3.04 to 3.69. Similarly, the titratable acidity of apple juices exhibited significant variability depending on the cultivar, ranging from 0.07 to 0.19 g/100 mL (expressed as malic acid).

The high water content and a_w_ levels confirm the inherent low microbiological stability of this type of food product. However, the application of pasteurization during production, combined with the low pH of the products and their refrigerated storage, ensures protection against microbial growth for an adequate shelf life [51]. These measures particularly inhibit the development of pathogens such as *Clostridium botulinum*, *Salmonella* spp., *Staphylococcus aureus*, *Listeria monocytogenes*, and *Escherichia coli* [26]. Nevertheless, due to their broad tolerance to low pH, reduced temperatures, and limited water activity, osmophilic yeasts and xerophilic molds may still pose a significant threat [52,53,54].

### 2.4. Phenolics Content and Antioxidant Activity

The total phenolic content (TPC), determined by the Folin–Ciocalteu method, ranged from 48.71 to 51.15 mg per 100 g of the sample (expressed as gallic acid equivalents) (Table 2). The products enriched with echinacea (FEP) exhibited the highest TPC value, differing significantly (*p* < 0.05) from the other samples. Conversely, the FSN and FAM product samples showed slightly lower polyphenol concentrations compared to the control (F0). Despite the data suggesting lower phenolic levels in FSN and FAM, it should be noted that this method is not highly selective, and the results may be overestimated due to the presence of non-phenolic reducing agents, such as fruit sugars (e.g., fructose). Consequently, the method utilizing the reaction with aluminum chloride in an alkaline medium is considered more selective and reliable for flavonoid determination. The total flavonoid content values obtained through this method were significantly higher in the fortified products compared to the control (F0), reaching a maximum of 0.103 mg/100 g for the echinacea-enriched product (FEP). The analyzed samples exhibited similar antioxidant activities when measured against ABTS and DPPH radicals. In the ABTS assay, the values fluctuated within a narrow range from 0.789 to 0.816 mM TE/100 g (Trolox equivalents) and showed no statistically significant differences (α = 0.05). However, in the DPPH assay, the echinacea-enriched product (FEP) and the control sample (F0) displayed significantly higher antioxidant activities than the other samples, with values of 0.226 and 0.219 mM TE/100 g, respectively (Table 2).

A strong linear correlation (r = 0.968) was found between the Folin–Ciocalteu and DPPH methods, suggesting that the reducing substances, including polyphenols identified spectrophotometrically, are primarily responsible for the antioxidant activity against the DPPH radical. The FRAP assay further confirmed the high reducing power of the echinacea-enriched product (FEP), which yielded the highest value among all samples at 0.692 mM/100 g Fe^+2^ (Table 2). The lowest reducing activity toward iron ions was observed in the elderberry-fortified product (0.587 mM/100 g). Significant linear correlation coefficients were observed between the FRAP results and those obtained via the Folin–Ciocalteu method (r = 0.9355) and the DPPH assay (r = 0.975). These findings indicate that the spectrophotometrically identified phenolic compounds in the analyzed fruit purée possess both antiradical and reducing properties.

In the methanol-water extracts of the analyzed samples, specific phenolic compounds belonging to the groups of phenolic acids and flavonoids were identified using high-performance liquid chromatography (HPLC) (Table 3). Two types of extracts were analyzed: non-hydrolyzed extracts, used to determine polyphenolic compounds occurring in their free form, and extracts subjected to alkaline hydrolysis. The latter allowed for the determination of phenolic compounds present as bound forms (i.e., esters and glycosides), which were degraded into their aglycone forms during the hydrolysis process. In the non-hydrolyzed extracts, one flavonoid was identified: catechin. Its concentration ranged from 22.159 mg/100 g (for F0) to 27.559 mg/100 g (for FEP). Catechins (flavanols) are unique among flavonoids as they do not typically form bound glycosides and occur primarily as aglycones, which explains their identification in the raw methanol-water extracts. The concentration of this flavonoid was more than 20 times higher than that of the other phenolic compounds identified in the samples.

Another flavonoid, hesperidin (a flavanone), was present in the samples in its bound form. The content of the free aglycone (hesperetin) ranged from 0.863 mg/100 g (for FSN) to 1.217 mg/100 g (for the control product). A small but statistically significant increase in catechin content was observed in the products enriched with fruit extracts, whereas no such trend was noted for hesperidin. This suggests that the primary source of hesperidin, and the dominant source of catechin, were the base fruits used to produce the control sample (F0). These observations may explain the lack of statistically significant differences in antioxidant activity measured via the ABTS assay, as well as the minimal decrease in antioxidant activity for FSN and FAM compared to the control. The exception was the FEP (enriched with echinacea), which achieved the highest values for DPPH radical scavenging activity, FRAP reducing power, and total flavonoid content (TFC). These results correlate with the notably higher catechin content determined by HPLC in the FEP sample compared to the others. Additionally, hydroxycinnamic acids (specifically caffeic and *p*-coumaric acids) were identified following their release from bound forms via hydrolysis. The caffeic acid content ranged from 0.843 to 1.610 mg/100 g, with the highest value recorded for the FEP product. While p-coumaric acid was most abundant in the control sample (F0), among the fortified samples, it reached its maximum concentration in the FEP sample.

Based on these chromatographic results, it can be concluded that the identified phenolic compounds originated primarily from the base fruits used in the control sample. However, among the fortified variants, the echinacea-enriched product (FEP) demonstrated the highest concentrations of the analyzed phenols (with the exception of p-coumaric acid). Consequently, the addition of echinacea significantly enhances the antioxidant and reducing properties of the fruit functional food.

### 2.5. Color Parameters

Table 4 presents the color parameters of the prepared fruit purée with oat flakes (Table 5). The color properties were expressed using three key descriptors: lightness (L*), hue angle (h*), and chroma (C*).

The incorporation of plant extract-based biocomposites significantly influenced the instrumental color parameters of the fruit functional foods (*p* < 0.05). All analyzed samples were characterized by medium lightness (L) values, which correspond to the average value reported for strawberry, banana, and apple purées [55,56,57]. The aronia-enriched product (FAM) showed the highest L* value (43.30), indicating increased brightness compared to the control (F0), while FSN and FEP remained closer to the control. All samples showed a predominance of red and yellow tones, as indicated by the positive a* and b* coordinates. A significant increase in a* values was observed for FAM and FEP, reflecting enhanced redness of these samples. Chroma (C*) values were higher in all enriched samples compared to F0, particularly in FAM and FEP, suggesting greater color saturation. The hue angle (h) ranged from 0.67 to 0.74, indicating a shift within the red–yellow region of the color space. The slightly higher h value in elderberries-enriched product (FSN) suggests a subtle shift toward more yellowish tones, whereas FEP exhibited a lower h, indicating a tendency toward deeper red coloration (Table 4). The darker color of the fruit products results not only from pigments naturally present in the fruits, but also from changes occurring in the raw material during technological processing, such as cutting, homogenization, and pasteurization. Mechanical homogenization enhances browning reactions as a result of significant tissue breakdown. The disruption of cellular compartments allows phenolic compounds from vacuoles to come into direct contact with polyphenol oxidase (PPO) located in plastids and the cytoplasm, thereby intensifying enzymatic browning. Moreover, the reduction in particle size increases oxygen exposure, which further promotes oxidative processes [58,59]. The browning susceptibility of banana and apple purée is largely determined by its chemical composition and enzyme activity [60]. The relatively soft structure of banana tissue, together with high moisture and sugar content, favors oxidation and enzymatic discoloration. On the other hand, the presence of organic acids, including citric and malic acids, may partially inhibit browning. These acids are capable of binding copper ions in the PPO active center, which reduces enzyme activity and limits the browning reaction [26,61,62,63]. In strawberries, reductions in color stability are primarily linked to the degradation of anthocyanins responsible for the fruit’s intense red pigmentation. Pelargonidin-3-glucoside and cyanidin-3-glucoside constitute the predominant anthocyanin fraction and play a decisive role in determining both the intensity and stability of the red hue; therefore, their degradation directly contributes to the observed color alterations [26,64].

The total color difference (ΔE*) values confirmed varying degrees of color changes among the fortified samples: FAM (3.20), FEP (2.07), and FSN (1.25) (Table 4 and Table 5). Samples with 1 < ΔE* < 2, including the elderberry-fortified purée (FSN), fall within a range where color variations are detectable only by trained or experienced observers [26]. Consequently, the visual distinction between FSN and the control is likely imperceptible to the average consumer. In contrast, it is generally accepted that a ΔE* value of approximately 2.0 or higher is necessary for a color difference to be clearly perceived by an untrained observer, which was the case for the FAM and FEP samples. However, the literature confirms that color deviations below a ΔE* threshold of 3.5 generally remain within acceptable industry tolerances for visual matching [65], which was the case for FAM and FEP samples. From a processing standpoint, maintaining the color parameters below this critical threshold demonstrates that these formulations can be enriched with bioactive compounds without drastically altering their traditional appearance, offering a significant industrial advantage.

### 2.6. Rheological Properties of Products

**Figure 3:**
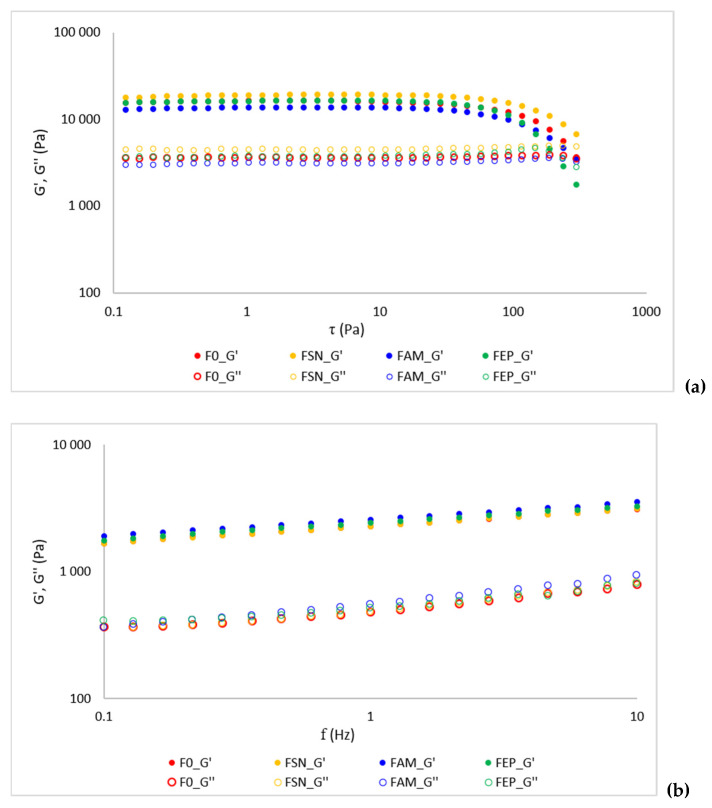
Dynamic mechanical spectra (G′, G″) of food products as a function of (**a**) stress (τ) and (**b**) frequency (f). F0—fruit purée with oat flakes with the addition of freeze-dried potato starch gel (control sample); FSN—fruit purée with oat flakes enriched with *Sambucus nigra* (elderberry) biocomposites; FAM—fruit purée with oat flakes enriched with *Aronia melanocarpa* (chokeberry fruit) biocomposites; FEP—fruit purée with oat flakes enriched with *Echinacea purpurea* (purple coneflower) biocomposites.

The results illustrate the dependence of the storage modulus (G′) and the loss modulus (G″) on the applied shear stress (0.1–300 Pa) (Figure 3) and varying frequency (0.1–10 Hz) (Figure 2) for the control fruit purée and variants enriched with encapsulated plant extracts. In all analyzed samples, the storage modulus (G′) exceeded the loss modulus (G″), confirming a predominance of elastic over viscous properties and the presence of a gel-like structure. Based on the curve profiles, the linear viscoelastic region (LVR) was identified, where the moduli remained nearly constant, indicating that the structure was undisturbed and stable under low stress [66,67].

Fruit purées are multiphase systems in which the continuous (aqueous) phase contains dispersed solid particles and air bubbles. The degree of structuring within the polysaccharide matrix—primarily involving pectins—plays a crucial role. Under appropriate conditions (pH, temperature, and the presence of sugars), pectins form a gel network responsible for resistance to deformation. A higher concentration of pectin-rich fruit in the product correlates with an increased capacity for gel network formation, which subsequently translates into higher hardness. In apples, pectins occur primarily in a high-methoxyl (HM) form (degree of methylation > 50%), which determines their gelation ability [68]. While limited changes in the degree of esterification occur during fruit ripening, significant demethylation is primarily driven by industrial processing or enzymatic activity, such as that of pectin methylesterase [69]. Thermal treatment promotes the degradation of fruit cell walls, leading to the breakdown and solubilization of pectins, thereby altering particle stiffness [70]. Consequently, heat processing may reduce the gelling capacity of pectins due to both partial demethylation and depolymerization of the polysaccharide chains, resulting in a weakened gel structure.

Another significant factor influencing the rheology of the analyzed system is the dry matter content, including sugars and fiber. These components affect the viscosity of the continuous phase and restrict water mobility, thereby increasing the rigidity of the system [71]. Furthermore, the inclusion of oat flakes contributes to structural stabilization due to the presence of β-glucans. These high-molecular-weight polysaccharides increase system viscosity by binding water and limiting its mobility [72,73].

The yield point represents the moment when the internal structure begins to undergo permanent damage (network breakdown), and the material transitions from a solid-like behavior to a more liquid-like state [66]. According to the data presented in Table 6, both the control and the fortified samples demonstrated relatively high τ_10%_ values, ranging from 37.89 to 53.96 Pa. Such results suggest the formation of a well-defined and mechanically robust internal network. The highest yield stress was recorded for the product enriched with encapsulated elderberry extracts, indicating its superior resistance to mechanical deformation and an increased tolerance to stress-induced structural breakdown. In contrast, the remaining formulations showed stability levels comparable to the control sample. Once the applied stress exceeded the LVR threshold, a sharp decline in G′ was noted, followed by a reduction in G″. This trend signifies a gradual collapse of the internal structure and a shift toward a more viscous, liquid-like response, with the F0 and FAM samples exhibiting the most significant susceptibility to this deformation.

The frequency dependence of the moduli, alongside the predominance of elastic over viscous properties, is characteristic of systems forming weak gels (Figure 3b) [66]. The rheological differences between the samples were minor, suggesting that the incorporation of capsules into the starch matrix does not compromise the rheological stability of the fruit products. These results also confirm the effectiveness of the delivery form used for the additives. It is noteworthy that the starch matrix encapsulated not only plant extracts but also grape seed oil. Had these additives been introduced directly into the fruit products, a noticeable decline in the product’s elastic properties would have occurred due to the presence of free oil—an effect confirmed in our previous studies [41]. Encapsulation diminishes the lubrication effect of the oil, allowing for controlled modulation of the textural and rheological properties of the fruit purées. Thus, the encapsulation technique not only shields bioactive substances from external factors but also facilitates the incorporation of diverse ingredients into food systems while maintaining the original stability of the matrix. Furthermore, the prepared capsule-containing matrices were subjected to lyophilization [39] before being incorporated into the fruit purées and pasteurized. From the initial formation of the capsules to the final product stage, they underwent a series of technological treatments without losing their structural integrity. Consequently, the starch–psyllium matrix serves as a robust shell, protecting the encapsulated substances from the fluctuating conditions encountered during food processing.

**Figure 4:**
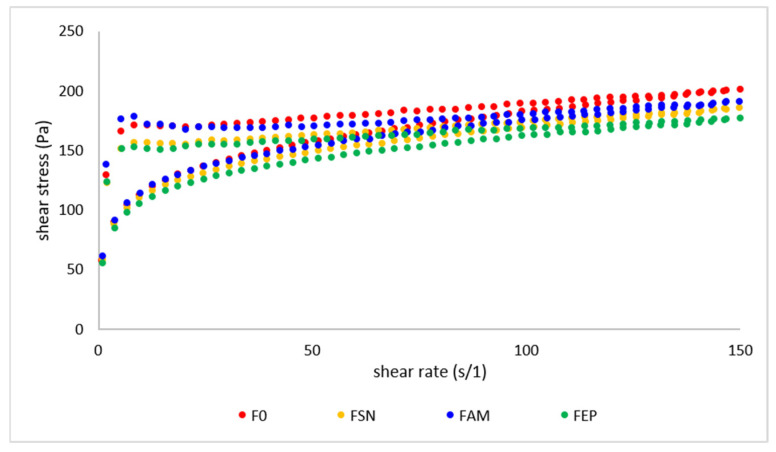
Flow curves of fruit purée with oat flakes. F0—fruit purée with oat flakes with the addition of freeze-dried potato starch gel (control sample); FSN—fruit purée with oat flakes enriched with *Sambucus nigra* (elderberry) biocomposites; FAM—fruit purée with oat flakes enriched with *Aronia melanocarpa* (chokeberry fruit) biocomposites; FEP—fruit purée with oat flakes enriched with *Echinacea purpurea* (purple coneflower) biocomposites.

The rheological properties of the formulation based on puréed fruits and oat flakes exhibited the behavior of non-Newtonian, shear-thinning fluids (Figure 4), similar to other fruit products such as pulps, purées, and mousses [71]. Although the Ostwald de-Waele model provided a good general fit for most samples (R^2^ > 0.93), a slightly lower coefficient (R^2^ = 0.825) was observed for the FAM product, which can be attributed to the highly complex microstructural interactions and pronounced yield stress behavior induced by the Aronia biocomposites (Table 7). The significantly higher consistency index values (>131 Pa·s^n^) of the formulated fruit formulation, compared to literature data reported for apple, banana, or strawberry purées [71], stem from the inclusion of oat flakes in the formulation. The oats largely dictate the high viscosity and consistency of the product, which subsequently impacts the mouthfeel and overall consumer acceptability. Aqueous beta-glucan solutions exhibit considerable viscosity, a high consistency index, and a flow behavior index below unity (*n* < 1), thereby displaying pseudoplastic (shear-thinning) fluid characteristics similar to fruit pulps [74]. Therefore, the rheological properties of the final product represent a composite outcome derived from each added ingredient. The resultant combination of these properties forms the product attributes perceived by the consumer, which can be tailored by adjusting the ingredient proportions. Correlating the rheological and textural properties with sensory evaluation facilitates the optimization of the formulation.

### 2.7. Texture Parameters of Products

The textural parameters of the formulated fruit purée with oat flakes are summarized in Table 8. All texture analyses were conducted on the freshly prepared samples. As previously discussed, the rheological and textural properties of fruit purée-based matrices are governed by a multitude of factors. The product’s microstructure, primarily driven by the degree of tissue fragmentation and homogenization, dictates the spatial arrangement and interactions of dispersed particles, which directly translate into the observed macroscopic textural attributes. Furthermore, these structural characteristics are significantly modulated by the selected processing conditions (e.g., thermal versus non-thermal treatments), as well as the specific composition and relative abundance of cell wall components, predominantly cellulose, hemicellulose, pectins, and structural proteins [75].

The minor differences in textural properties among the samples indicate that fortification with encapsulated plant extracts and grape seed oil did not significantly alter the structural integrity of the product. Although the hardness of the formulated products was higher than that of typical fruit purée [26,41], it remained relatively consistent across all variants, ranging from 1.17 to 1.23 N. The presence of oats in the formulation resulted in a more robust texture, significantly increasing the firmness of the otherwise delicate fruit purées. Most texture attributes, specifically adhesiveness and springiness, showed no significant differences. However, slight discrepancies were found in cohesiveness when comparing control sample (F0) with FAM and FEP, while differences in gumminess occurred between the control and all fortified variants. The increase in cohesiveness and gumminess observed in the fortified samples suggests that the incorporated capsules act as active fillers within the polysaccharide matrix of the fruit purée. Strong adhesion between the capsule surfaces and the continuous phase components (e.g., pectins and β-glucans) likely promotes a reinforcement effect, enhancing the overall structural integrity and resistance to disintegration during mechanical deformation [76].

The highly effective encapsulation process enabled the successful entrapment of both the plant extracts and the grape seed oil within the polysaccharide shell. Furthermore, the structural stability of the capsules was fully preserved even after the pasteurization process.

### 2.8. Microbiology

#### 2.8.1. Microbiological Stability During Storage

The microbiological analysis of fruit purée enriched with encapsulated extracts from chokeberry, elderberry, and echinacea showed exceptional stability throughout the 60-day storage period. In all tested variants, no microbial growth was observed at any of the analyzed time points (days 0, 30 and 60). Specifically, the total bacterial count (TBC), total yeasts and mold count (TYMC), as well as the tested indicator and pathogenic microorganisms (*E. coli*, *Salmonella* spp., *S. aureus* and *Enterococcus* spp.) remained below the detection limit (<1 CFU). The observed microbiological stability can be attributed primarily to the combined effect of pasteurization, low pH of the product (4.08–4.11) and the content of polyphenols: flavonoids and phenolic compounds which are known to exhibit a variety of antimicrobial mechanisms [77]. The effect of acidic pH on microbiological stability of food products is among the established food microbiology principles. Acidic environments and the presence of organic acids in the food products limit the survival and proliferation of food spoilage microorganisms, e.g., *Clostridium botulinum*, *Salmonella* spp., *Listeria monocytogenes* and *E. coli* [78]. These results indicate that the pasteurization process and the inherent properties of the fruit-extract compositions effectively prevented microbial proliferation, ensuring the microbiological safety of the functional products for at least two months of storage at room temperature.

#### 2.8.2. Antibacterial Activity of Fruit Purée

The antibacterial properties of the extract-enriched fruit purée were evaluated against a broad panel of 58 bacterial isolates, including 33 Gram-positive and 25 Gram-negative strains.

##### Analysis of Bacterial Susceptibility Between Gram Type

**Figure 5:**
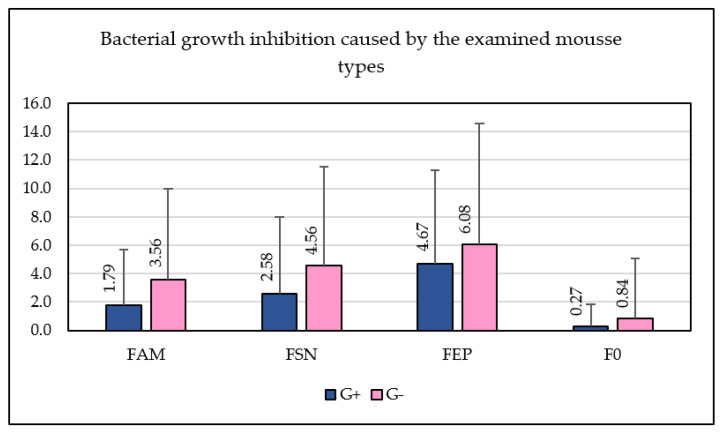
Antibacterial effects of the examined fruit products with plant extracts (FAM—*Aronia melanocarpa*, FSN—*Sambucus nigra* and FEP—*Echinacea purpurea*) and control samples (F0), exhibited by the growth inhibition caused by the application of the fruit purée on top of the agar inoculated with Gram-positive (n = 33) and Gram-negative (n = 25) bacteria. Error bars show standard deviations.

The overall efficacy of the tested formulations against Gram-positive (G+) and Gram-negative (G−) bacteria is presented in Figure 5 and Table 9. A consistent trend was observed across all enriched products, where Gram-negative bacteria exhibited higher susceptibility compared to Gram-positives. Furthermore, differences in the samples enriched with different extracts were observed. FEP (enriched with echinacea) demonstrated the highest overall activity, with mean inhibition zones of 6.08 mm for G− and 4.67 for G+ isolates. It was followed by FSN (enriched with elderberry) with mean inhibition zones of 4.56 mm for G− and 2.58 for G+ and FAM (with chokeberry) which caused mean zones of 3.56 mm for G− and 1.78 for G+. Also, some antibacterial activity of F0 (control) was observed with mean values below 1 mm for both G− and G+. The relatively high standard deviations (error bars in Figure 5) reflect the significant biological diversity among the reaction of all 58 tested isolates. Interestingly, the observed clear difference in susceptibility between Gram-positive and Gram-negative bacteria was noted in the present study, with Gram-negative strains exhibiting higher susceptibility to all extract-enriched fruit purée. This pattern mirrors our previous findings obtained for the corresponding encapsulated plant extracts tested solely [39]. This indicates that the incorporation of encapsulated extracts into the fruit purée matrices did not alter their antibacterial selectivity. Although Gram-negative bacteria are often described as less susceptible to antimicrobial agents than Gram-positive ones, due to the presence of a lipopolysaccharide-rich outer membrane that acts as a permeability barrier, several phenolic acids (identified in the examined formulations) have been shown to destabilize the outer membrane, increase its permeability, resulting in leakage of intracellular compounds [39,79]. Also, the acidic pH of the fruit purée could act synergistically with these phenolic compounds, further increasing cell membrane permeability. According to Cotter and Hill [80], Gram-positive bacteria are much more resistant to acidic pH than Gram-negatives, due to a variety of mechanisms, including efficient proton pumps, protection or repair of macromolecules, cell membrane changes, alteration of metabolic pathways or biofilm formation. Such low pH as the one encountered in the fruit purée could first compromise the cells of Gram-negative bacteria which then become more susceptible to the effect of active compounds present in fruit purée. Importantly, the increasing antibacterial activity observed across our formulations (FAM < FSN < FEP) reflects the increasing concentration of phenolic acids in the formulations which may suggest that the compound composition is the primary factor affecting the antibacterial effectiveness.

The control sample (F0) exhibited a baseline inhibitory effect only against two strains, i.e., Gram-negative *Pseudomonas koreensis* (21 mm) and Gram-positive *S. aureus* (single strain, 9 mm of inhibition). However, the addition of encapsulated extracts significantly enhanced this effect, with the FEP variant increasing the inhibition zone to 29 mm. This suggests a potential additive or synergistic interaction between the fruit-derived organic acids and the secondary metabolites of encapsulated plant extracts.

##### Species-Specific Response

**Figure 6:**
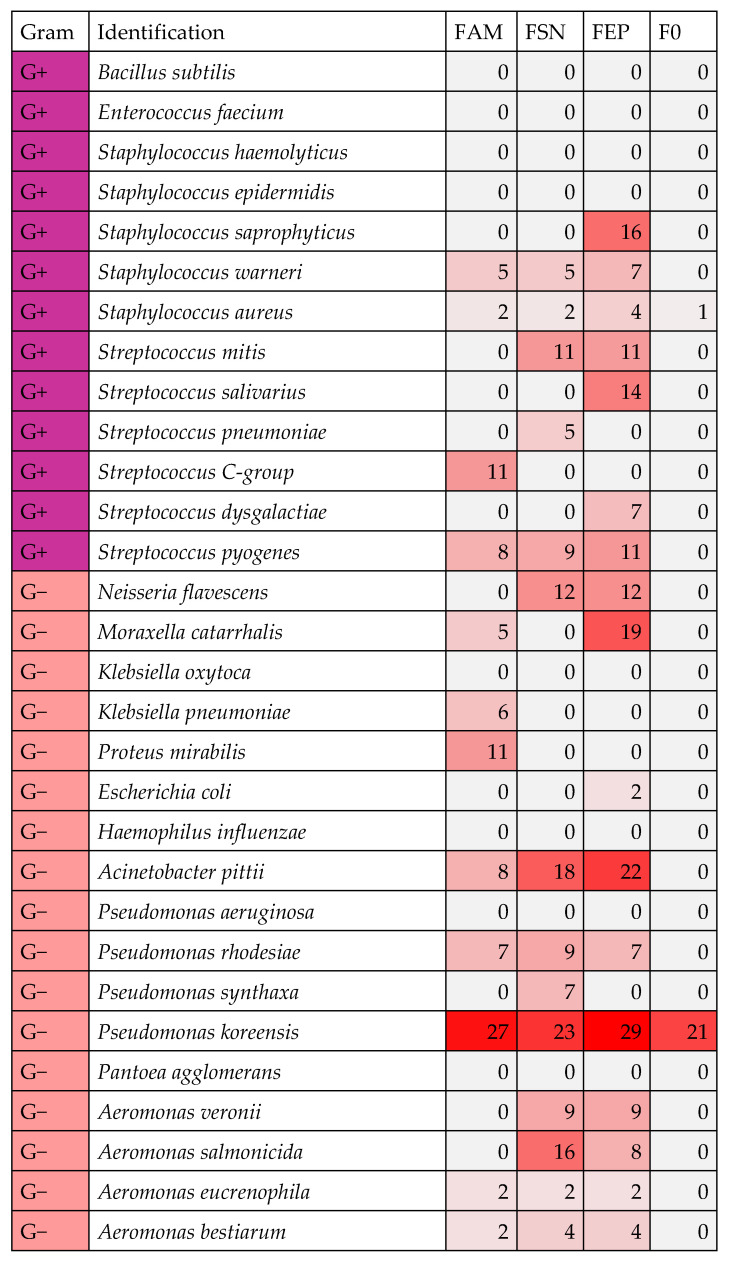
Heatmap of the mean growth inhibition zones (mm) of fruit purée enriched with encapsulated extracts (FAM: chokeberry; FSN: elderberry; FEP: echinacea; F0: control) against the examined bacterial species. Data represent the mean values for all isolates within a given species. Color scale: pale gray (0 mm) to dark red (maximum inhibition).

To further examine the antimicrobial spectrum, the results were aggregated as mean inhibition zone diameters for each bacterial species and showed in a heatmap (Figure 6). The complete data for all 58 isolates are summarized in Supplementary Table S1.

The most susceptible Gram-negative pathogens included: *Moraxella catarrhalis* (mean zone of 19 mm for FEP formulation), *Acinetobacter pittii* (22 mm for FEP) and *Neisseria flavescens* (12 mm for FSN and FEP). Interestingly, *Pseudomonas koreensis*, an environmental microorganism, was highly susceptible to all formulations with growth inhibition zones reaching 29 mm for FEP, as well as growth inhibition caused by a control purée.

**Figure 7:**
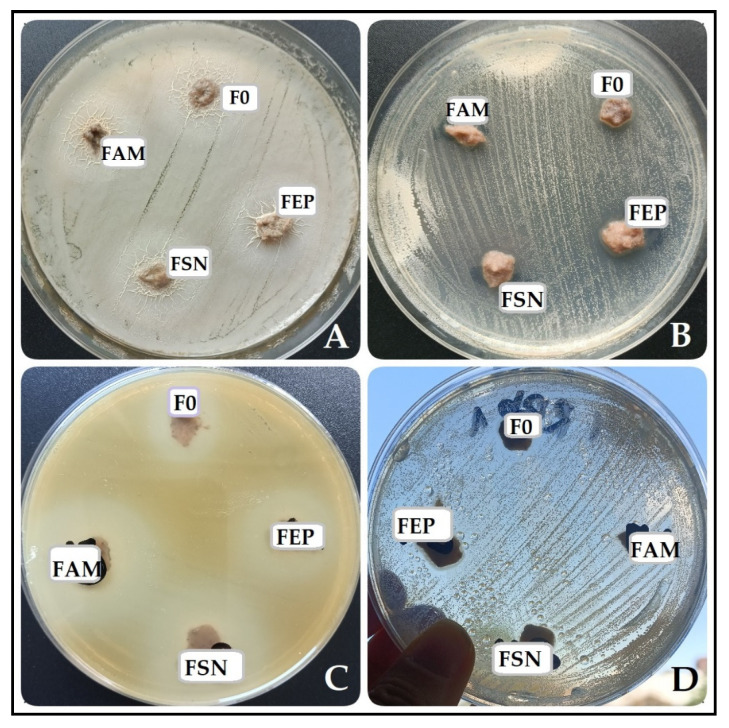
Photographs showing the variety of bacterial reactions to the application of the developed formulations. (**A**) *Bacillus subtilis*; (**B**) *Aeromonas bestiarum*; (**C**) *Pseudomonas koreensis*; (**D**) *Staphylococcus aureus*.

Among Gram-positive potential pathogens, the highest susceptibility was observed for *Streptococcus salivarius* (14 mm for FEP) and *Streptococcus mitis* (11 mm for FSN and FEP). The growth of individual *S. aureus* strains was inhibited, particularly by FEP (growth inhibition zones up to 14 mm, Supplementary Table S1). However, out of 12 *S. aureus* isolates, the growth of five was inhibited by the application of the examined formulations—the others remained resistant. Therefore, the mean growth inhibition presented in Figure 7 ranged from 1 to 4 mm.

Importantly, one-third (n = 11) of bacterial species examined in this study were represented by more than one isolate, while two thirds (n = 19) occurred only once. In the case of *S. aureus*, a total of 12 isolates were examined and the inter-species variation in susceptibility to the applied formulations was significant (Supplementary Table S1). For example, while some *S. aureus* isolates remained completely unaffected by the activity of the fruit purée, others exhibited substantial inhibition zones (up to 14 mm). Also, one of the *Streptococcus mitis* strains was characterized by some of the largest inhibition zones (22 mm for FSN, 21 for FEP), while the second one showed no reaction. The fact of strain-to-strain variance, its effect on the antimicrobial efficacy examination results and underappreciation was discussed by Sanchez and Martinez [81] or Nysten et al. [82]. Such inter-species differences in microbial reactions to various compounds that are intended to limit their growth and proliferation stem from, e.g., the ability of an isolate to form biofilms (which form mechanical barriers for compound penetration), individual resistance mechanisms that may result from previous exposure to antimicrobials, individual virulence factors, as well as microenvironmental parameters. With this in mind, further examinations—if intended to strictly assess the antimicrobial potential of the developed formulations—should be focused on a limited number of different species and broader panel of the species’ strains.

However, what should be pointed out here is the fact that even though the antibacterial effect (understood as growth inhibition or growth rate reduction) was not the primary aim when designing the fruit purée, it clearly appears from the data that the addition of encapsulated fruit extracts to the plant base significantly increased its antibacterial potential. The growth of 36.4% Gram-positive isolates and 44% Gram-negative isolates was inhibited by the application of the FEP (enriched with echinacea), followed by FSN (enriched with elderberry), which inhibited the growth of 21.2% of Gram-positive isolates and 27.6% of Gram-negatives (Table 9). As discussed in our earlier study [39], the strong antibacterial effect of *Echinacea purpurea* extracts results from its rich content of phytochemicals such as caffeic acid derivatives, β-caryophyllene, α-pinene, germacrene D, α-phellandrene, β-pinene, γ-curcumene, and δ-cadinene. The antibacterial effect of these compounds has been demonstrated in vitro [83] and confirmed in our experiments.

### 2.9. Analysis of Volatile Compounds Profile Using Headspace Method on Electronic Nose

The qualitative and quantitative analysis of volatiles profile determined using the electronic nose (HERACLES2/Neo) enabled the detection and identification of volatile compounds (Table 10) belonging to the several groups of organic compounds including: aldehydes, alcohols, esters of organic acids, organic acids, terpenoids, and a sulfur-containing compound (methional).

The first group of volatile compounds present in both oat flakes and the fruits used for samples preparation consists of aldehydes (Table 10). Their total content ranged from 14.77% of the volatile fraction for sample FEP to 18.10% for sample FAM. Among them, the following compounds were identified: acetaldehyde, hexanal, and methional, with hexanal occurring in the highest amounts among the aldehydes, ranging from 12.93% for sample FEP to 15.87% for sample FAM.

Hexanal is one of the key volatile compounds, contributing to aroma formation in the oat flakes [87,88]. The six-carbon aldehydes (including hexanal) are synthesized as a result of the oxidation of linoleic and linolenic acids present in cereal grains through the activity of lipoxygenase [102]. Besides oat flakes, the source of hexanal in the studied samples also includes the fruits used in their formulation, i.e., apples [93], strawberries [89,90], and bananas [91,92]. High content of hexanal in the volatile fraction under studied samples (Table 10) may contribute significantly to their characteristic aroma profile. This aldehyde is associated with fruity, grassy, and herbal aroma notes, while its odor threshold in air is very low, amounting to 0.005 mg/m^3^ (Table 11), which indicates its important role in shaping the aroma of all analyzed samples. The addition of chokeberry and elderberry lyophilizates (in case of FAM and FSN samples) did not significantly increase hexanal content in those samples compared to the control one (F0), indicating that hexanal is not a major volatile component of the applied fruit additives in the form of lyophilization.

The second most abundant aldehyde, present in the analyzed samples was acetaldehyde (Table 10), with concentrations ranging from 1.84% for sample FEP to 2.23% for sample FAM. The presence of this aldehyde has also been reported in apples [84], strawberries [85] and bananas [86], which were used in the production of all the analyzed samples. Acetaldehyde contributes to aromas perceived as apple-like, fruity, or floral (Table 11), and it is characterized by a low odor threshold value of 0.08 mg/m^3^. Trace amounts of methional (<0.1%) (a sulfur-containing aldehyde that was identified earlier in oat grains) were detected in samples F0 and FSN, respectively.

The analyzed samples also proved to be a significant source of alcohols (Table 10), among which ethanol reached the highest values, ranging from 10.66% for sample FEP to 16.21% for sample FAM. The presence of ethanol as a volatile compound has been earlier identified in bananas [86,91] and strawberries [85]. Ethanol, being the second most abundant volatile component, may be responsible for the characteristic: ethereal, pleasant, and sweet aroma (Table 11). However, due to its relatively high odor recognition value (190 mg/m^3^), its contribution to the aroma formation of analyzed samples may be lower when compared with the aldehydes and esters present in significant amounts in the analyzed samples (Table 10 and Table 11).

The alcohols occurring in the analyzed samples in the small amounts (appr. 1%), were 2-methyl-1-propanol present in all the analyzed samples and 2-methyl-1-butanol, present only in sample FAM at a level of 1.12%.

Among the identified alcohols, linalool (Table 10) was characterized by the lowest odor threshold value (0.002 mg/m^3^), whereas for heptan-2-ol and 2-methyl-1-butanol these values were much higher: 0.14 and 0.10 mg/m^3^, respectively. Regarding 2-methyl-1-propanol, its presence was reported in banana fruits [86,95], on the other hand, 2-methyl-1-butanol was one of many alcohols occurring in apples [84,96]. Despite the significantly lower concentrations of the mentioned above alcohols compared to ethanol, these compounds may have a greater influence on the aroma profile of the analyzed samples than ethanol itself, particularly linalool characterized by the lowest odor threshold, present in the sample FAM, since this alcohol is characterized by floral, spicy or citrus aroma notes (Table 11). In contrast, heptan-2-ol, present in most samples, contributes balsamic, pungent and cheese-like aroma notes (Table 11). The presence of this alcohol has been earlier reported in the volatile fraction of bananas [86,91,97].

2-Methyl-1-butanol was detected only in sample FAM (Table 10) and may be responsible for balsamic, banana-like, and wine-like aroma notes. 2-Methyl-1-propanol is characterized by a relatively high odor threshold value (2.32 mg/m^3^), and due to its low concentration (approximately 1%), it is unlikely to play a significant role in shaping of aroma of the studied samples.

Propan-2-one (acetone) was the only ketone identified in the analyzed samples (Table 10), with its level ranging from 1.50% for sample FSN to 1.92% for the control sample (F0). Based on the obtained data (Table 10), it can be concluded that its main source was the fruits used for the preparation of the control sample (F0) rather than the applied lyophilizates (in case of remaining samples). This ketone is characterized by a relatively high odor threshold value (480 mg/m^3^) and apple, fruity and sweet aroma (Table 11). The presence of acetone as a volatile compound has previously been identified in the volatile fraction of strawberries [85].

Esters constituted the group of organic compounds present in the highest amounts in analyzed samples (Table 10), with their total content (in percentage) in all the samples being similar, amounting to approximately 40% of the volatile fraction above analyzed samples. The esters occurring in the highest concentrations included: ethyl acetate, butyl acetate, hexyl acetate and isoamyl acetate (Table 10).

Ethyl acetate is a very widespread ester commonly present in fruits. Its content in the analyzed samples ranged from 11.39% for sample FEP to 16.34% for sample FAM. Its presence has previously been identified in apples [84,93,96], bananas [91], and strawberries [85]. It is responsible for the characteristic aroma notes of many fruits and is described as: ethereal, fruity, and pineapple-like (Table 11). However, its odor threshold is relatively high (>5 mg/m^3^).

Butyl acetate and isoamyl acetate are the volatile compounds responsible for highly characteristic banana-like, fruity, sweet, and pleasant aromas, with isoamyl acetate additionally resembling the aroma of apples and pears (Table 11). The content of butyl acetate ranged from 8.91% (for FEP) to 9.82% (for FSN), whereas isoamyl acetate was present in the volatile fraction at levels ranging from 4.58% (for FAM) to 10.77% (for FEP). Since isoamyl acetate and butyl acetate are characterized by the lower odor threshold values than ethyl acetate (0.14 and 0.7 mg/m^3^ versus 5 mg/m^3^, respectively), they should contribute much more significantly than ethyl acetate to the aroma formation of analyzed samples. In particular, the differences may be noticeable in the aroma of sample FAM compared to the remaining samples due to high differences in isoamyl acetate and ethyl acetate contents (Table 10). Butyl acetate has previously been identified in bananas [91,95,100], apples [93], and strawberries [90].

Hexyl acetate was another abundant aroma ester, with its content ranging from 3.41% for sample FAM to 4.69% for sample FSN (Table 10). Since it is characterized by an even lower odor threshold value (0.08 mg/m^3^) when compared with mentioned above esters, its presence may significantly influence the aroma of the analyzed samples, imparting: sour, fruity, herbal, or spicy odor notes (Table 11). This ester is widely found in fruits, and was found in banana [86], strawberry [85,90] and apple fruits [84,93,96].

Among the six-carbon esters, ethyl hexanoate was identified in the majority of analyzed samples, with concentrations ranging from 1.30% for sample (F0) to 2.31% for sample FAM. The low odor threshold value of this ester (0.03 mg/m^3^) may contribute to the characteristic anise-like, banana-like, and fruity aroma notes (Table 11) recognized in the analyzed samples. Similarly to hexyl acetate, ethyl hexanoate is also widespread in various fruits, and was also detected in bananas [91,92], strawberries [98,99,103] and in apples [93,101].

Another group of esters, identified in the analyzed samples consisted of derivatives of 2-methylbutanoic acid (Table 10), present in the form of methyl and ethyl esters. These esters are responsible for characteristic fruity and apple-like aroma notes, while the ethyl ester additionally contributes strawberry-like notes. Although the methyl ester of 2-methylbutanoic acid occurred in the higher amounts in the analyzed samples, the ethyl ester, with a very low odor threshold value of 0.0012 mg/m^3^, contributes much more significantly to the aroma formation compared to the methyl ester, whose odor threshold is 1.18 mg/m^3^. Ethyl 2-methylbutanoate is responsible for apple, fruity, sharp and strawberry aroma and is present in strawberries [90] and bananas [95], whereas the presence of methyl 2-methylbutanoate was reported in apples [84,96] and strawberries [85,89].

The content of ethyl 2-methylbutanoate ranged from 1.11% for sample FAM to 1.88% for sample FEP. The remaining esters, namely ethyl heptanoate, ethyl octanoate, and pentyl octanoate, were present in trace amounts in the analyzed samples (Table 10). Ethyl heptanoate was detected only in the control sample (F0), accounting for 0.18% of the volatile fraction above the sample. This ester is characterized by a fruity aroma and an odor threshold value of 0.19 mg/m^3^.

Ethyl octanoate occurred in trace amounts in all the samples, ranging from 0.14% (for FSN) to 0.27% (for FEP); however, it is characterize by low odor threshold (0.02 mg/m^3^); thus, it may be responsible (in particular in FEP sample) for anise-like, floral, fruity, or menthol-like aroma odor. Presence of this ester was earlier reported in strawberries [90,98,100]. Pentyl octanoate was present in most of the analyzed samples in trace amounts at approximately 0.05%.

The presence of pentanoic acid at a level of 0.35% was detected in the volatile fraction above the sample enriched with chokeberry (FAM). This acid is characterized by a low odor threshold value (0.01 mg/m^3^) and by a sharp, pungent, cheesy, and rancid odor. The presence of low amounts this acid was reported in bananas [95].

The last group of volatile compounds identified in the analyzed samples consisted of terpenoids (Table 10). Among these class of compounds, β-damascenone may be considered as sensory-significant minor component due to its extremely low odor threshold value (0.00004 mg/m^3^), which means that despite its low concentrations, it may be responsible for aroma differences between the sample FEP (containing this compounds), and the remaining ones due to characteristic β-damascenone aroma. This terpenoid may be characterized by fruity, apple-like, honey-like, or rose-like aroma notes depending on its concentration in air. Myrcene possesses a characteristic balsamic, fruity, ethereal and musty aroma, and its odor threshold is 0.37 mg/m^3^. Myrcene is a crucial volatiles present in the various parts of *Echinacea* [102,103]. Despite the low concentrations of these terpenoids, they may distinguish the aroma of sample FEP from the other analyzed samples, which is demonstrated in the further part of this manuscript by using principal component analysis (PCA). On the contrary, α-terpineol was identified in only two analyzed samples FSN and FEP; however, FEP sample was again the richest source of this compound. α-Terpineol is responsible for floral, fruity, mint-like, or peach-like aroma notes, and its odor threshold value is 0.19 mg/m^3^. The presence of this terpenoid was reported by the several authors in strawberries [99] and apples [104].

**Figure 8:**
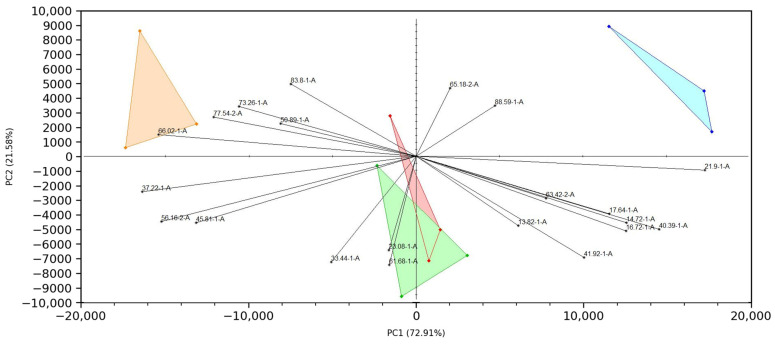
The results of PCA for the studied samples including F0, FSN, FAM and FEP taking into account 3 replications (i.e, 01–03) for each sample. The numerical value at a given point indicates the retention time [min]; the designation 1-A corresponds to the retention time obtained on MXT-5 column, whereas 2-A corresponds to MXT-1701 column, respectively. The letter A indicates the relative peak area of a given peak subjected to PCA. The triangles forming the three replication were marked in different colors: red for F0, green for FEP, blue for FAM, and orange for FSN.

To group the studied samples in terms of type and amounts of identified volatile compounds (Table 10), a PCA was performed (Figure 8) on the basis of retention times of particular volatile compounds originating from the first column (MXT-5) or the second one (MXT-1701). The amounts of volatiles subjected to this statistical analysis were expressed as relative amounts (areas of the chromatographic peaks) in relation to the total area of all the detected peaks for the analyzed sample.

The analytical variables (i.e., contents of individual volatile compounds) of the samples under study were processed using a principal component analysis (PCA) in order to show grouping of the studied samples. The PCA explained 93.40% of the analytical variables, with 71.65% and 21.76% explained by the first and second factors, respectively (Figure 8).

Each analyzed sample was presented on the plot in the form of a triangle corresponding to three repetitions of the sample analysis. The projection of the four samples was presented on the plot surface defined by Factors I and II. The application of PCA enabled grouping of the individual analyzed samples according to the types of volatile compounds and their relative amounts in the volatile phase in the sample headspace.

On the plot, the first group (formed by the samples FSN and F0), located in the lower part of the graph (Figure 8), can be observed to exhibit a clear similarity in terms of the profile of volatile compounds. These samples may best be characterized by the presence of substances negatively correlated with Factor II, and among them, particularly 2-methyl-1-propanol, 2-methyl-1-butanol, and hexyl acetate. Among these volatile compounds, especially hexyl acetate and 2-methyl-1-butanol are characterized by particularly low odor threshold values (0.08 and 0.14 mg/m^3^, respectively) (Table 11). All of these substances share certain sensory descriptors, such as banana-like, fruity and vinous aromas, and in the case of hexyl acetate also herbal/green aroma notes, which may distinguish these two samples (i.e., F0 and FSN) from the others.

Another sample, clearly distinguished from the others, is sample FEP, located in the upper right region of the plot. This sample may best be characterized by the terpenoid β-damascenone, which despite its low concentration in the sample is characterized by a very low odor threshold value (0.00004 mg/m^3^), which may also contribute to aroma differences. Depending on its concentration in the volatile phase, β-damascenone is responsible for fruity, honey-like, apple-like, or rose-like aroma notes. Additionally, α-terpineol, with a low odor threshold value (0.19 mg/m^3^), may contribute to the distinct aroma of this sample, described as fruity, citrus-like, minty, anise-like, or peach-like. This sample may also be well characterized by the relatively high amount of heptan-2-ol (0.82%). The presence of heptan-2-ol, an alcohol with a pungent and raw odor, also characterized by a low odor threshold value (0.10 mg/m^3^) is also notable. Additionally, two esters including ethyl heptanoate and ethyl octanoate may characterize that sample, since they are present in the highest amount among all the samples tested. In particular, ethyl octanoate characterized by a low odor threshold (0.02 mg/m^3^) may be responsible for the characteristic anise, floral, fruity, leafy and mentholic aromas. Regarding low-molecular-weight esters, also ethyl acetate, present in the volatile fraction above the sample in the amount of nearly 17%, may be responsible for ethereal, fruity, pineapple and sweet aromas; however, the odor threshold (recognition) is not so low (5.08 mg/m^3^).

The use of the electronic nose and PCA allowed for a precise, instrumental validation of the volatile profile, directly corresponding with the specific aroma notes identified later by the sensory panel.

### 2.10. Sensory Profile of the Developed Functional Food

**Figure 9:**
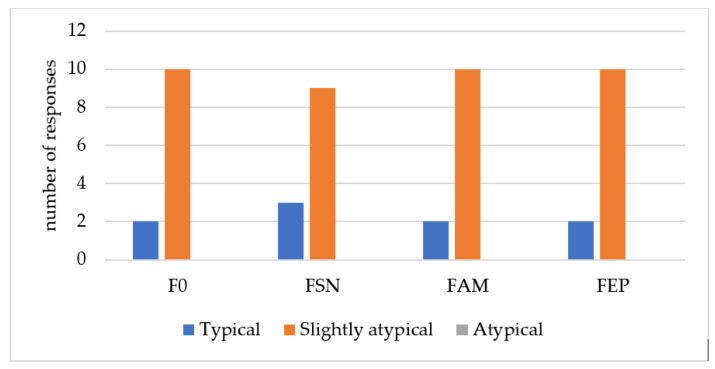
Sensory analysis of the external appearance of fruit products.

**Figure 10:**
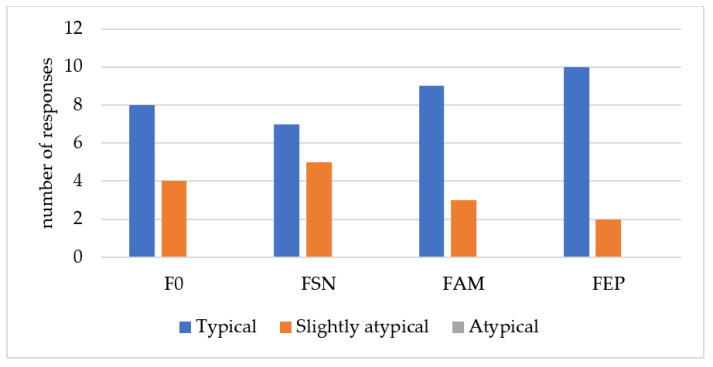
Sensory analysis of the color of fruit products.

**Figure 11:**
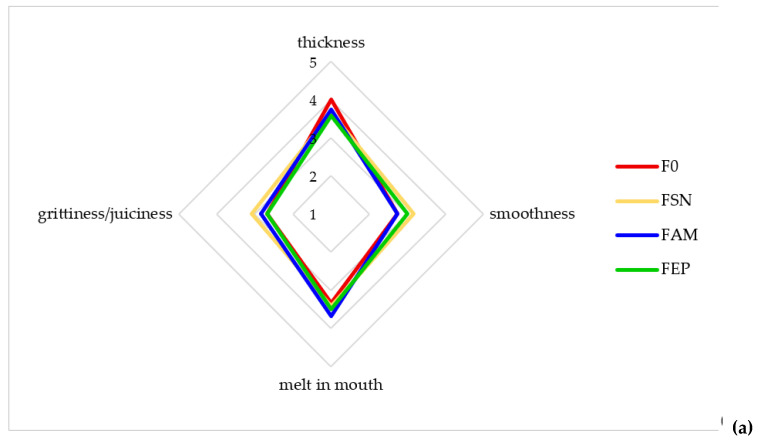

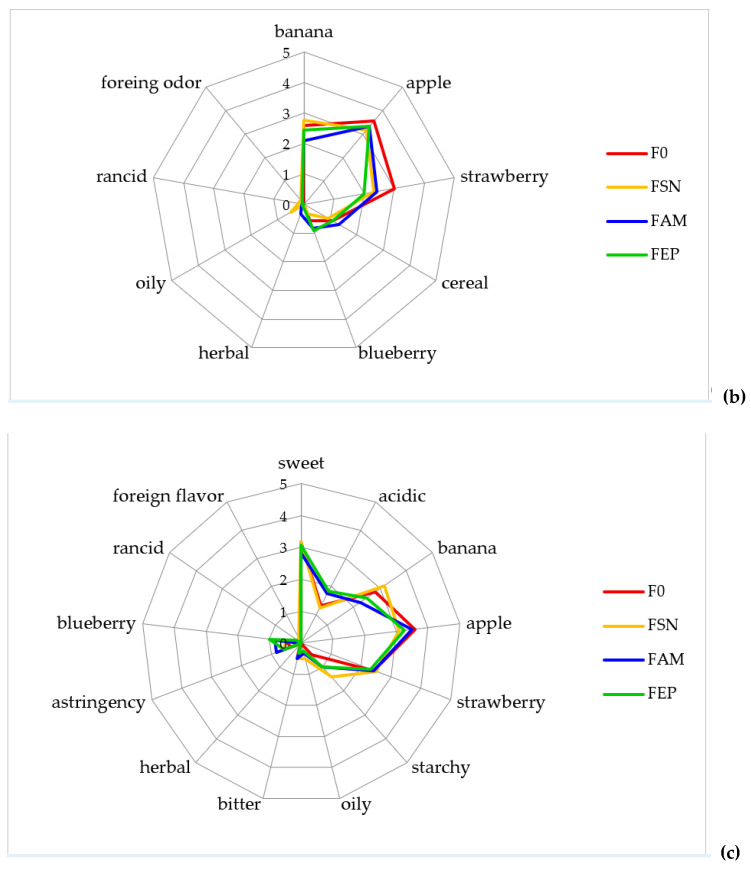
Sensory analysis of the (**a**) texture, (**b**) aroma and (**c**) taste profile of fruit products.

All fruit purée variants, including the control sample (F0) and those fortified with encapsulated plant extracts (FSN, FAM, FEP), were evaluated for their sensory properties using Quantitative Descriptive Analysis (QDA). The obtained results are presented in Figure 9, Figure 10 and Figure 11. Initially, the sensory panel evaluated the external appearance of the analyzed products. The assessment focused on surface smoothness, overall consistency, phase stability (absence of phase separation), and the potential occurrence of syneresis (water exudation). All evaluated variants received similar scores, exhibiting high structural stability with no signs of stratification. Due to the localized presence of minor, visible solid particulates originating from the raw materials, the samples were assigned the descriptor “slightly atypical”.

It should be emphasized that despite the incorporation of an oil component in the fortified purée, its successful entrapment within the capsule shell effectively prevented structural destabilization, oil phase separation, and the occurrence of syneresis.

The majority of the panelists described the color of the products as “typical” (Figure 10). Echinacea-enriched product (FEP) was primarily rated as “typical”, whereas for the remaining samples, some panelists assigned the descriptor “slightly atypical” due to minor opalescence and color heterogeneity, which can be observed in fruit products containing various ingredients. All products exhibited a color within the red-yellow spectrum, as indicated by the h* parameter derived from instrumental L*a*b* color measurements, leading the panel to perceive them as slightly pink. The color of the products was characteristic of the raw materials used (i.e., apple, banana, and strawberry). However, the addition of the extract induced a slight change that was noticeable to the panelists. This observation was further corroborated by the instrumentally determined ΔE* values. The most pronounced color differences among the products were observed between the F0 sample and both the FAM and FEP variants.

The evaluated textural attributes included thickness, smoothness, melt-in-mouth, and grittiness/juiciness. As illustrated in the graph (Figure 11a), no significant differences were observed among the samples regarding these characteristics. Similarly, the instrumental texture analysis revealed no statistically significant differences among the products for the majority of the evaluated parameters.

Intense apple, strawberry, and banana aroma notes, along with a less pronounced cereal note, were identified through sensory analysis across all samples (Figure 11b). The presence of volatile chemical compounds responsible for these fruity and cereal aromas was also detected using the electronic nose (Section 2.9). The dominant aroma note perceived by the panelists was apple, which is attributed to the highest percentage of this ingredient in the final product (60%). In the F0 sample, the strawberry aroma was perceived more intensely than in the other products; however, this observation could not be confirmed by the electronic nose analysis. It should be noted that a specific aroma can be attributed to several different chemical substances, as previously discussed in this paper. Furthermore, the perceived intensity of a given note does not necessarily indicate a high concentration of the corresponding compound, since chemical compounds exhibit varying odor detection thresholds in air. Regarding the remaining aroma notes, the panel found no significant differences among the samples. The cereal aroma was slightly more intense in the FAM product compared to the FEP variant, which correlates with a higher percentage of hexanal—the primary volatile component responsible for the oat flake-derived odor.

The evaluated products exhibited similar flavor profiles (Figure 11c). Minor differences among the samples were noted in their perceived acidity. The FEP sample was characterized by the highest perceived acidity, which was corroborated by the total acidity measurements (Table 1). In the fortified products, the panelists detected a very faint oily note, along with slightly bitter and astringent flavor notes. Additionally, the starchy note was slightly more pronounced than in the control sample (F0). Overall, the differences in the flavor profiles among the samples were minimal. Furthermore, no rancid or off-notes were detected in either the aroma or flavor profiles, which would have otherwise disqualified the products in terms of quality.

### 2.11. Consumer Acceptability

**Figure 12:**
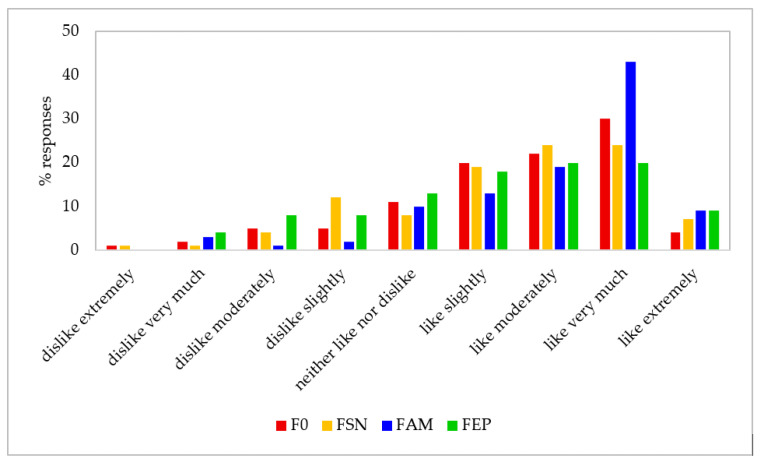
Consumer evaluation of the overall acceptability of the products.

Consumer evaluation confirmed the high quality of the fortified fruit products (Figure 12). Some consumers awarded slightly lower scores due to an unsatisfactory consistency, specifically the presence of lumps and perceptible oat flake fragments. Conversely, the most frequently cited positive attributes were mild acidity and moderate sweetness, which consumers associated with the naturalness of the product (the formulation contained no added sucrose, and the perceived sweetness was derived entirely from naturally occurring sugars in the fruits). A slight perception of oiliness was reported sporadically; this observation was noted by one individual for the FEP sample and two for the FSN variant. Additionally, a few panelists reported astringency when evaluating the products containing encapsulated chokeberry, echinacea, and elderberry extracts. However, it should be emphasized that the vast majority of consumers perceived no significant differences among the evaluated variants.

Consumers were asked to rank the samples according to their preference, assigning ranks from 1 (least preferred) to 4 (most preferred). The resulting rank sums for each product are summarized in Table 12. To evaluate the statistical significance of the differences among the products, a Friedman test was applied in accordance with the ISO standard [105]. The null hypothesis (*H*_0_) assumed no difference in consumer preference among the samples, while the alternative hypothesis (*H*_1_) postulated that a significant difference exists between at least two products. The calculated Friedman test statistic (F_test_ = 2.92) was lower than the critical tabular value (F_crit_ = 7.81) for the given number of assessors and products at a predefined significance level of α = 0.05. Consequently, there was no basis to reject the null hypothesis. It can therefore be concluded that there are no statistically significant differences in consumer preference among the evaluated variants.

Ultimately, the positive sensory feedback validates the entire fortification strategy, proving that the physical and chemical stability of the microcapsules—as demonstrated by instrumental analyses—successfully translated into a high-quality consumer product.

## 3. Materials and Methods

### 3.1. Materials

The base ingredients of the fruit purée with oat flakes were strawberries (*Fragaria* × *ananassa*), ripe bananas (*Musa acuminata*), apples (*Malus domestica*, cultivar Prince), and oat flakes purchased from the local market (Kraków, Poland). Biocomposites containing extracts from dry elderberry fruit (*Sambucus nigra* L.), dry chokeberry fruit (*Aronia melanocarpa*), and dry purple coneflower (*Echinacea purpurea*) were prepared according to an original methodology developed at the Faculty of Food Technology, University of Agriculture in Krakow, and described in detail in our previous paper [39]. Briefly, the biocomposites consisted of encapsulated plant extracts and grape seed oil. Capsule stabilization was achieved by immobilizing them within a polysaccharide matrix composed of psyllium mucilage gel and potato starch gel. The obtained emulsions were subjected to freeze-drying to yield dry biocomposite preparations, the initial properties of which were comprehensively characterized in our preliminary research [39], which confirmed their structural and functional viability through SEM, FTIR, thermal properties, wettability, and antioxidant activity assessments. 

### 3.2. Preparation of Fruit Purée with Oat Flakes

The formulation for the fruit purée with oat flakes and the processing conditions were optimized in preliminary experiments. Fruits (strawberries, bananas, apples) were peeled, cut into small pieces, and then blended with oat flakes into a homogeneous mixture using a kitchen blender (Götze & Jensen, Copenhagen, Denmark) for 2 min. The mixture was then cooked for 10 min. The base product was enriched with additional ingredients (Table 13), depending on the fruit purée with oat flakes variant:

- F0—fruit purée with oat flakes, with the addition of freeze-dried potato starch gel (control sample).
- FSN—fruit purée enriched with *Sambucus nigra* (elderberry) biocomposites.
- FAM—fruit purée with oat flakes enriched with *Aronia melanocarpa* (chokeberry fruit) biocomposites.
- FEP—fruit purée with oat flakes enriched with *Echinacea purpurea* (purple coneflower) biocomposites.

After thoroughly mixing all the ingredients, the product was transferred into small glass jars, which were then pasteurized in hot water for 15 min. The pasteurization of the final fruit product served a dual purpose: ensuring microbiological stability and completely inactivating native polyphenol-oxidizing enzymes (such as PPO and POD), thereby protecting the introduced encapsulated bioactives from enzyme-mediated degradation. Once cooled, the samples were stored under refrigerated conditions at 4 °C.

### 3.3. Scanning Electron Microscopy (SEM)

The morphology of the dried encapsulated samples was examined using a JEOL 7550 scanning electron microscope (JEOL Ltd., Akishima, Tokyo, Japan). To enhance conductivity, the sample was sputter-coated with a 20 nm layer of chromium using a K575X Turbo Sputter Coater (Emitech Ltd., Ashford, UK).

### 3.4. Fourier-Transform Infrared Spectroscopy (FTIR)

The FTIR spectra were acquired for all dried samples. A MATTSON 3000 FT-IR spectrophotometer (Madison, WI, USA) equipped with a MIRacle ATR accessory (PIKE Technologies Inc., Madison, WI, USA) was employed. Spectra were collected at 20 °C (±2 °C) across a wavenumber range of 4000–700 cm^−1^.

### 3.5. Moisture Content

The moisture content of the samples was determined using the drying oven method. For this purpose, 1 g of sample was placed in a MAC 50 moisture analyzer (Radwag, Radom, Poland) and dried until a constant weight was achieved. Each measurement was performed in duplicate for each sample variant.

### 3.6. Water Activity

Water activity was measured using an AquaLab 4TE device (Decagon Devices, Inc., Pullman, WA, USA) with an accuracy of ±0.0003. Measurements were performed at a temperature of 20 °C (293 K) in duplicate for each sample.

### 3.7. Determination of pH

The pH of the fruit purée with oat flakes was determined using a PH-100 ATC pH-meter (Voltcraft, Hirschau, Germany) by measuring the electromotive force, which was used to calculate the potential of the sample.

### 3.8. Total Acidity

The titratable acidity (TA) was determined by alkalimetric titration, which involves neutralizing the acids present in the sample with a standardized alkaline solution. The analysis was performed in accordance with the Polish Standard PN-A-75101-04:1990 [106].

### 3.9. Polyphenols and Antioxidant Activity

#### 3.9.1. Procedure for Extraction of Phenolic Compounds from the Analyzed Samples

In order to extract the phenolic compounds from the samples under study, approximately 10 g of sample was weighed and transferred into a 100 mL volumetric flask. The flask was then filled with an aqueous-methanol solution (80:20, *v*/*v*) to the volume of approximately 60 mL. The resulting mixture was extracted at room temp. for 24 h. Then, the content of the flask was filled up to 100 mL with the extracting solution. The resulting extract was defatted in a separatory funnel by triple extraction with petroleum ether. The defatted extract was subsequently subjected to liquid chromatography (HPLC). Before this step, the extract was earlier purified using a solid-phase extraction (SPE). The purified extract was directly subjected to the liquid chromatograph (HPLC).

#### 3.9.2. Determination of Total Phenolic Content in the Analyzed Samples

Total phenolic content (TPC) was determined using Folin–Ciocalteu’s method [107]. Briefly, 0.5 mL of the extract was mixed in a probe with 2.5 mL of diluted (1:10, *v*/*v*) Folin reagent. After a few min of incubation, 2 mL of a 7.5% sodium carbonate solution was added to each probe. The blank sample was prepared using deionized water instead of extract. After 2 h of incubation, an absorbance of the solution was measured at λ = 760 nm against blank sample. The results were expressed in mg of gallic acid per 100 g. The calculations were based on the calibration curve based on gallic acid solutions.

#### 3.9.3. Determination of Total Flavonoid Content in the Tested Samples

Total flavonoid content (TFC) was determined using the method developed by Barnum et al. [108]. For this aim, 1 mL of the extract was mixed in a probe with 4 mL of water. Then, 0.3 mL of a sodium nitrite solution, followed by 0.3 mL of aluminum chloride solution, and finally 4 mL of sodium hydroxide solution was added mixing the solution after each reagent addition. The final volume of reaction solution was then adjusted to 10 mL with water. A blank sample was prepared in the same way, replacing the extract with water. The results were expressed in quercetin equivalents (mg) per 100 g. The calibration curve is based on quercetin solutions.

#### 3.9.4. Determination of Antioxidant Activity Using a DPPH Assay of the Analyzed Samples

Antioxidant activity (AA) of the samples was determined using the method developed by Blois [109]. Briefly, 0.1 mL of the extract was mixed in a tube with 3.0 mL of DPPH radical solution. After 60 min of incubation, an absorbance was measured at λ = 515 against methanol as a blank sample. The results were expressed in mM of trolox per 100 g. The calculations were based on the calibration curve using trolox solutions.

#### 3.9.5. Determination of Antioxidant Activity Using an ABTS Assay of the Analyzed Samples

Antioxidant activity (AA) was determined using method developed by Baltrušaitytė et al. [110]. Briefly, 0.1 mL of the extract was mixed in a probe with 6.0 mL of ABTS^+•^ solution, appropriately diluted with the phosphate buffer. After 30 min of incubation, an absorbance of resulting solution was measured at λ = 734 against the phosphate buffer as a blank sample. The obtained results were expressed in mM of trolox per 100 g. The calculations were based on the calibration curve which was made using trolox solutions.

#### 3.9.6. Determination of Reducing Activity of the Samples Using a FRAP Assay

Reducing activity of the samples was determined using method developed by Benzie and Strain [111]. Briefly, 3.3 mL of acetate buffer was mixed in a tube with 0.33 mL of 20 mM ferric chloride solution following by 0.33 mL of 10 mM TPTZ solution in hydrochloric acid and finally was incubated at the temp. of 37 °C. After the end, 0.33 mL of the extract was added to the obtained solution following by the further incubation for 15 min. After cooling, absorbance was measured at λ = 593 nm. The results were expressed as mM of ferrous ions (Fe^2+^) per 100. The calculations were based on the calibration curve equation which was made using FeSO_4_ solutions.

#### 3.9.7. Procedure for Analysis of Free Phenolic Compounds in the Tested Samples

The chromatographic analysis of extracts was carried out using a high-performance liquid chromatograph (Jasco, Tokyo, Japan) equipped and diode array detector (DAD, Jasco, Japan). The presence of hydroxybenzoic acids was identified at 280 nm, and hydroxycinnamic acids were identified at 320 nm, whereas flavonoids including catechin and hesperidin were identified at a λ = 280 nm. Chromatographic analysis was carried out using a reversed phase (RP-18) column (Purospher, Darmstadt, Germany). The gradient elution chromatography was employed using the two mobile phases: phase A (2.5%, (*m*/*v*), acetic acid solution in water) and phase B (acetonitrile of gradient grade), based on the earlier described procedure [112]. Qualitative analysis was made by comparing the retention times of chromatographic peaks with those of standards as well as by comparing the absorption (UV) spectra of the detected peaks with corresponding spectra of the standards. Quantitative analysis was made using the calibration curves prepared for the analyzed phenolics. The chromatographic analyses were performed in triplicate.

#### 3.9.8. Procedure for Analysis of Bound and Free Forms of Phenolic Compounds in Tested Samples

In order to determine the level of phenolics occurring in bound forms in the samples under study, extracts were subjected to an alkaline hydrolysis according to the method developed by Nardini and Ghiselli [113]. Briefly, 10 mL of the extract was mixed with 90 mL of a hydrolyzing solution. The resulting solution was incubated at 30 °C. After completion of alkaline hydrolysis, the solution was cooled to r.t. and then acidified with diluted hydrochloride acid until pH = 2 was reached.

The extraction of phenolics released was performed according to procedure described by Socha et al. [114]. For this aim, the acidified phenolics solution was saturated with sodium chloride until saturation was reached. Subsequently, the obtained solution was extracted three times with ethyl acetate. After the end of extraction, the collected ethyl acetate fractions were combined and evaporated using a vacuum rotary evaporator residue was obtained. The resulting solid residues were dissolved in 5 mL of methanol and were stored at 5 °C until the chromatographic analysis. Before a HPLC analysis, the methanolic extracts were purified by a solid-phase extraction (SPE), analogously to the method applied for the samples not subjected to hydrolysis.

### 3.10. Color Measurement

The color of the food products was evaluated using a Konica MINOLTA CM-3500d spectrophotometer (Konica Minolta Inc., Tokyo, Japan), equipped with a 30 mm diameter measuring aperture and employing D65 illumination with a 10° standard observer. Color parameters were recorded in the CIELab color space, including L*, a*, and b*. The L* value represents lightness, ranging from 0 (black) to 100 (white). The a* value reflects the red-green axis, with negative values indicating green and positive values indicating red. Similarly, the b* value corresponds to the blue-yellow axis, where negative values denote blue and positive values denote yellow.

Each measurement was repeated five times, and the final result was calculated as the mean of these values. From the recorded data, additional color parameters: chroma (C*), hue angle (h*), and total color difference (ΔE*), were also determined [39].

Saturation (chroma, C*) describes the intensity of a color in relation to a neutral gray of the same lightness. Higher C* values indicate more vivid and intense colors as perceived by the human eye.

$$C^{*}=\sqrt{{a^{*}}^{2}+{b^{*}}^{2}}$$

The hue angle (h*) represents the position of a color on the three-dimensional color wheel, with typical reference points being 0° for red, 90° for yellow, 180° for green, and 270° for blue, as perceived by the human eye.

$$h^{*}={tan}^{-1}\left(\frac{b^{*}}{a^{*}}\right)$$

The total color difference (ΔE*) represents the color difference between the samples and the standard (control sample).

$${\Delta E}^{*}=\sqrt{{\Delta a}^{*2}+{\Delta b}^{*2}+{\Delta L}^{*2}}$$

### 3.11. Rheological Measurement

The rheological properties were assessed based on the method described by Krystyjan et al. [26], with slight modifications. Measurements were performed using a RheoStress RS 6000 rotational rheometer (Thermo Scientific, Karlsruhe, Germany) equipped with a plate–plate PP60 Ti system (diameter 60 mm, gap 2 mm). The temperature of the bottom plate was maintained at 20.0 ± 0.1 °C. Analyses were conducted on freshly prepared samples, and each measurement was carried out in triplicate.

Oscillation stress sweep test: Linear viscoelastic region (LVR) of emulsions were determined in the range 0.1–300 Pa at constant frequency (1 Hz). The linear viscoelastic region (LVR, plateau) and the yield point were determined from the storage modulus G′ profile. The yield point was calculated as the stress at which the material no longer exhibited a linear viscoelastic response and G′ decreased by 10% relative to the plateau value. This 10% criterion is a conventional threshold recommended by standard [115] and supported by literature data [116].

Frequency sweep test: Viscoelastic properties of samples were measured in the range 0.1–10 Hz at 1 Pa deformation fitting the range of linear viscoelasticity. The results for storage modulus (G′) and loss modulus (G″) were recorded [67].

Flow curves: The shear rate was raised from 0.1 to 100 s^−1^, over a 5 min period and a subsequent decrease in shear rate from 100 to 0.1 s^−1^, over a 5 min. Obtained flow curves were described by Ostwald de-Waele rheological model:

$$\tau =K\cdot {\dot{\gamma }}^{n}$$

where *τ*—shear stress (Pa), *K*—consistency coefficient (Pa·s^n^), $\dot{\gamma }$—shear rate (s^−1^), and *n*—flow behavior index.

### 3.12. Texture Analysis

The textural characteristics of the fruit purée were evaluated using a TA.XTplus texture analyzer (Stable Micro Systems Ltd., Godalming, Surrey, UK). A Texture Profile Analysis (TPA) test was conducted with a cylindrical probe (20 mm diameter), operating at a speed of 1 mm/s. The probe performed a double compression to a strain 50% under a constant load of 2.5 g. Samples were placed in jars with a diameter of 55 mm, and measurements were carried out at 20 ± 0.5 °C. Force–distance curves generated during the test were used to calculate key textural parameters, including: hardness (N), adhesiveness (N·s), cohesiveness (dimensionless), and chewiness (N) [39]. Analyses were conducted on freshly prepared samples, and each measurement was carried out in five replicates.

### 3.13. Microbiological Analyses

*Analysis of microbiological stability of fruit purée*

Microbiological analyses were performed at three distinct time intervals to evaluate the shelf-life and stability of the products and these were: day 0, immediately after pasteurization; day 30, after one month of storage; and day 60, after two months of storage.

Microbiological quality of the products was assessed using the spread plate method. One loopful of samples were aseptically spread on the surface of general and selective microbiological media. The following parameters were determined: total bacterial count (TBC; incubated at 36 ± 1 °C from 24 h to 7 days), total yeast and mold count (TYMC; incubated at 25 ± 1 °C from 3 to 10 days). The following pathogens were also detected and enumerated: *Escherichia coli* (Tryptone-Bile-X-Glucuronide Agar, incubation at 44 °C for 24 h), *Salmonella* spp. (XLD and SS agar, incubation at 36 ± 1 °C for 24 h), *Staphylococcus aureus* (Baird-Parker agar, incubation at 36 ± 1 °C for 24–48 h) and *Enterococcus* spp. (Slanetz-Bartley agar, incubation at 36 ± 1 °C for 48–72 h).

*Analysis of antibacterial potential of fruit purée*

The antibacterial activity of fruit purée was evaluated against a total of 58 bacterial isolates (from the culture collection of the Department of Microbiology and Biomonitoring, University of Agriculture in Krakow): 33 Gram-positive strains and 25 Gram-negatives. Species identification of the bacterial isolates was previously determined by the MALDI-TOF MS method.

Bacterial suspensions were prepared in sterile saline solution (0.85% NaCl) and adjusted to an optical density of 0.5 McFarland (referring to c.a. 1.5 × 10^8^ CFU/mL). The prepared inoculum was then uniformly spread on the surface of Mueller–Hinton Agar (MHA) plates to create a bacterial lawn.

Equal amounts of the fruit products were applied onto the inoculated agar surface using a calibrated loop (10 µL). The samples were deposited as uniform spots with a diameter of approx. 5 mm. To facilitate the controlled release of bioactive constituents from the capsules, a two-step incubation protocol was implemented. Firstly, a pre-incubation at room temperature (22 ± 2 °C) for 24 h to allow the diffusion of the extracts into the medium before the rapid growth of bacteria. This was followed by standard incubation, when the plates were transferred to an incubator and maintained at 36 °C for 24–28 h to promote the bacterial growth.

After the incubation period, the plates were examined for the presence of clear zones of growth inhibition around the purée spots. The antibacterial activity was analyzed by measuring the diameters of growth inhibition zones (if present; including the 5 mm spot). All tests were performed in triplicate and the results were expressed as the mean diameter (mm) ± standard deviation.

### 3.14. Determination of Volatile Compounds and Odor Profile of the Tested Sample Using E-Nose

The qualitative and quantitative analysis of volatile compounds present in the tested sample was performed using a HERACLES2/Neo electronic nose (Alpha MOS, Toulouse, France) using the software (AlphaSoft v16, Alpha MOS, Toulouse, France). The determination of volatiles profile was conducted using the headspace method followed by the separation of volatile compounds on two independent chromatographic columns: non-polar (MXT-5), and semi-polar (MXT-1701) using a gas chromatography method with detection by two flame ionization detectors (FID-1 and FID-2). In order the qualitative analysis of volatiles separated chromatographically, the calibration was applied in the first step. For this aim, the calibration was carried using the standard consisting of hydrocarbons mixture (C6–C16). On the basis of Kovat’s retention index values obtained for the particular components of hydrocarbons mixture, it is possible to identify the volatile substances by comparing their Kovat’s retention index values of the chromatographic peaks with those obtained for the hydrocarbons mixture (C6–C16) by using software (AroChemBase v7, Alpha MOS, Toulouse, France). In order to differentiate the studied samples by means of type and amount (i.e., relative area) of the volatile compounds, principal component analysis (PCA) was performed using the software AlphaSoft v16.

In order to analyze volatile compounds, 2.0 g of the tested sample was weighed in a 20 mL glass vial that was crimped by the magnetic cap, and subsequently was subjected to gas chromatographic analysis. The injection volume was 5000 µL, injection temperature was 200 °C, and syringe temperature was equal 60 °C. The incubation temperature (before analysis) was equal to 50 °C, and time of incubation lasted 20 min. During the chromatographic separation, the linear gradient of temperature was applied, namely the initial oven (containing two columns) was equal to 80 °C and increased linearly form 80 °C to 250 °C in the range of time from 0 to 21 s. Hydrogen was used as a carrier gas, and the split flow was 10 mL/min. The temperature of FID detectors was 260 °C. All the samples were analyzed in three independent replicates. The level of individual volatile compounds was given as a relative area of chromatographic peak (%) in relation to the summary area of all peaks detected for the individual sample under study.

### 3.15. Sensory Analysis

The sensory analysis of the fruit products was carried out on the second day after they were produced. The analyses were performed by twelve experienced judges, trained in accordance with ISO sensory standards (ISO 3972:2011, ISO 4120:2021, ISO 5492:2008, ISO 5495:2005, ISO 8586:2023, ISO 11132:2021) [117,118,119,120,121,122] in individual booths with uniform lighting conditions at the Laboratory for Sensory Analysis in Centre for Innovation and Research on Prohealthy and Safe Food, University of Agriculture in Krakow, designed in accordance with the ISO 8589:2007 standard [123].

The Quantitative Descriptive Analysis (QDA) method (ISO 13299:2016) [124] was employed to assess the intensity of the sensory attributes. Initially, the panel leader, in collaboration with a select group of technologists and experienced sensory assessors, generated a preliminary list of descriptors. Through open discussion, the most relevant and frequently cited descriptors were selected. Clear definitions were established for each descriptor, and a structured 0–5 point scale, anchored at both extremes, was implemented. The panelists were instructed to comprehensively evaluate the following sensory modalities of the formulated products: appearance, texture, aroma, and taste (descriptor definitions are provided in Table S1 of the Supplementary Materials). The results were visualized using bar charts and radar (star) diagrams, where the intensity of each attribute was plotted as the distance from the center.

The research activities regarding sensory analysis were approved by the University of Agriculture Ethics Committee (Approval No. 330/2025, 6 October 2025) and all participants provided informed consent.

### 3.16. Consumer Testing

The consumer testing was conducted in the sensory laboratory at the Centre for Innovation and Research on Prohealthy and Safe Food (University of Agriculture in Krakow). The study involved a panel of 106 consumers: 73 women, 32 men, and 1 non-binary individual. All participants were of legal adult age, with the following age distribution: 18–24 years (n = 42), 25–34 years (n = 16), 35–44 years (n = 12), 46–55 years (n = 28), and over 56 years (n = 8). The samples were served in odorless, lidded plastic containers and coded with randomized three-digit numbers to ensure a blind assessment, concealing which sample was the control and which contained the encapsulated extract. Spring water was provided to the panelists between sample tastings for palate cleansing. All samples were served at room temperature. One part of the evaluation included a ranking test, conducted in accordance with the ISO 8587:2006 standard [105]. Participants were asked to rank the samples according to their preference, assigning ranks from 1 (least preferred) to 4 (most preferred). The rank data were subsequently analyzed using the non-parametric Friedman test to determine whether statistically significant differences existed among the evaluated variants at a significance level set at α = 0.05. Furthermore, consumers were asked to rate their overall liking of the products using a 9-point hedonic scale, ranging from 1 (“dislike extremely”) to 9 (“like extremely”). Participants were also provided with the opportunity to express open-ended verbal or written comments regarding the evaluated products.

### 3.17. Statistical Analysis

The data were subjected to statistical analysis using one-way analysis of variance (ANOVA) followed by Fisher’s test (*p* < 0.05) to determine significant differences between the tested sample variants. The data obtained from the consumer ranking test (rank sums) were analyzed using the non-parametric Friedman test to determine statistically significant differences among the samples, with the significance level set at α = 0.05.

## 4. Conclusions

The formulated product aligns with the clean-label trend by excluding all synthetic additives. Instead of chemically modified hydrocolloids, it utilizes natural biopolymers (psyllium and potato starch) for structural stability. Furthermore, safety and fortification are achieved through standard thermal processing and the addition of natural plant biocomposites, completely eliminating the need for artificial preservatives. Combined SEM and ATR-FTIR analyses demonstrate that these carriers provide a structurally stable, chemically inert protective shell. By forming non-covalent, hydrogen-bonded microstructures with naturally released fruit pectins during thermal processing, the carrier successfully isolates sensitive bioactive compounds and unsaturated fatty acids without disrupting the native food matrix.

The comparable total polyphenol content and antioxidant activity between the control and fortified variants (FSN, FAM, FEP) further validate the efficacy of this physical barrier. The tight entrapment and extensive hydrogen bonding with the carrier prevented the full release of bioactives during standard in vitro extraction. This indicates successful protection against premature release in the food matrix, thereby preserving their bioactivity for targeted gastrointestinal delivery.

Macroscopically, the integration of the biocomposites did not impair the product’s native framework. Textural and rheological parameters remained identical to the control, and visual acceptability was fully preserved with negligible color shifts (ΔE < 3.5). Concurrently, volatile profiling and sensory evaluations confirmed that the fundamental fruity profile remained dominant. The carrier effectively masked undesirable extract flavors, allowing trace compounds like terpenoids to introduce unique, highly acceptable aromatic nuances, resulting in no statistically significant differences in consumer preference.

Finally, the fortified products maintained a safely low pH despite high moisture content. This physicochemical stability, combined with the encapsulated extracts of elderberry, chokeberry, and echinacea, resulted in a microbiologically stable product with moderate bacteriostatic properties, particularly against Gram-negative bacteria and select oral microbiota strains. Ultimately, these findings highlight the immense potential of the developed starch–psyllium matrices in designing high-quality functional foods with extended shelf-life, exceptional sensory appeal, and targeted protective health effects.

While this study successfully establishes the structural, rheological, and sensory viability of the fresh product alongside its initial microbiological safety, further long-term storage trials under real-world conditions are required to fully evaluate textural stability and the degradation kinetics of the encapsulated bioactive compounds over time.

## Figures and Tables

**Table 1:** Basic parameters of products.

Sample	Water Content (%)	Water Activity (a_w_)	pH	Total Titratable Acidity (g Malic Acid/100 g)
F0	79.66 ± 0.09 ^b^	0.93 ± 0.00 ^a^	4.08 ± 0.01 ^b^	0.31 ± 0.01 ^b^
FSN	79.28 ± 0.25 ^b^	0.93 ± 0.00 ^a^	4.11 ± 0.01 ^a^	0.29 ± 0.01 ^c^
FAM	80.28 ± 0.56 ^a^	0.93 ± 0.00 ^a^	4.09 ± 0.01 ^b^	0.31 ± 0.00 ^b^
FEP	79.72 ± 0.14 ^ab^	0.93 ± 0.00 ^a^	4.10 ± 0.00 ^ab^	0.36 ± 0.01 ^a^

The values are expressed as the mean ± standard deviation. The presence of the same superscript letter (a–c) in each column indicates that there is no statistically significant difference between the values (*p* < 0.05). F0—fruit purée with oat flakes with the addition of freeze-dried potato starch gel (control sample); FSN—fruit purée with oat flakes enriched with *Sambucus nigra* (elderberry) biocomposites; FAM—fruit purée with oat flakes enriched with *Aronia melanocarpa* (chokeberry fruit) biocomposites; FEP—fruit purée with oat flakes enriched with *Echinacea purpurea* (purple coneflower) biocomposites.

**Table 2:** Total polyphenol and flavonoid content, and antioxidant and reducing activity of the tested samples.

Sample	Total Phenolic Content mg GAE/100 g	Total Flavonoids Content mg QE/100 g	DPPH Assay mM TE/100 g	ABTS Assay mM TE/100 g	FRAP Assay mM Fe (II)/100 g
F0	50.614 ^b^ ± 0.669	0.064 ^a^ ± 0.007	0.219 ^b^ ± 0.002	0.816 ^a^ ± 0.014	0.645 ^c^ ± 0.012
FSN	49.071 ^a^ ± 0.433	0.088 ^b^ ± 0.001	0.210 ^a^ ±0.068	0.809 ^a^ ± 0.005	0.587 ^a^ ± 0.051
FAM	48.714 ^a^ ± 0.795	0.065 ^a^ ± 0.011	0.211 ^a^ ±0.024	0.803 ^a^ ± 0.022	0.616 ^b^ ± 0.012
FEP	54.149 ^c^ ± 1.176	0.103 ^c^ ± 0.001	0.226 ^b^ ±0.003	0.789 ^a^ ± 0.021	0.692 ^d^ ± 0.008

The values are expressed as the mean ± standard deviation. The presence of the same superscript letter (a–d) in each column indicates that there is no statistically significant difference between the values (*p* < 0.05). GAE—gallic acid equivalents; QE—quercetin equivalents; TE—trolox equivalents; Fe (II)—ferrous ions equivalents; mM—milimoles. F0—fruit purée with oat flakes with the addition of freeze-dried potato starch gel (control sample); FSN—fruit purée with oat flakes enriched with *Sambucus nigra* (elderberry) biocomposites; FAM—fruit purée with oat flakes enriched with *Aronia melanocarpa* (chokeberry fruit) biocomposites; FEP—fruit purée with oat flakes enriched with *Echinacea purpurea* (purple coneflower) biocomposites.

**Table 3:** Content of phenolic compounds present in free and the sum of free and bound forms determined by chromatographic analysis.

Sample	Catechin	Caffeic Acid	*p*-Coumaric Acid	Hesperedin
F0	22.159 ^a^ ± 0.580	—	—	—
FSN	22.868 ^a^ ± 0.530	—	—	—
FAM	24.950 ^b^ ± 0.553	—	—	—
FEP	27.559 ^c^ ± 0.520	—	—	—
F0	22.159 ^a^ ± 0.580	1.054 ^c^ ± 0.018	1.609 ^c^ ± 0.038	1.217 ^d^ ± 0.026
FSN	22.868 ^a^ ± 0.530	0.843 ^a^ ± 0.008	1.478 ^a^ ± 0.023	0.863 ^a^ ± 0.010
FAM	24.950 ^b^ ± 0.553	0.933 ^b^ ± 0.008	1.431 ^a^ ± 0.023	1.033 ^b^ ± 0.012
FEP	27.559 ^c^ ± 0.520	1.610 ^c^ ± 0.008	1.506 ^b^ ± 0.007	1.144 ^c^ ± 0.030

The values are expressed as the mean ± standard deviation. The presence of the same superscript letter (a–d) in each column indicates that there is no statistically significant difference between the values (*p* < 0.05). F0—fruit purée with oat flakes with the addition of freeze-dried potato starch gel (control sample); FSN—fruit purée with oat flakes enriched with *Sambucus nigra* (elderberry) biocomposites; FAM—fruit purée with oat flakes enriched with *Aronia melanocarpa* (chokeberry fruit) biocomposites; FEP—fruit purée with oat flakes enriched with *Echinacea purpurea* (purple coneflower) biocomposites.

**Table 4:** Color parameters of fruit purée with oat flakes.

Sample	L* (D65)	a* (D65)	b* (D65)	C*	h*	ΔE*
F0	41.01 ± 0.02 ^d^	14.09 ± 0.03 ^d^	12.14 ± 0.04 ^d^	18.60 ± 0.04 ^d^	0.71 ± 0.00 ^c^	-
FSN	41.90 ± 0.02 ^b^	14.31 ± 0.02 ^c^	12.99 ± 0.04 ^b^	19.32 ± 0.02 ^c^	0.74 ± 0.00 ^a^	1.25
FAM	43.30 ± 0.04 ^a^	15.65 ± 0.08 ^b^	13.73 ± 0.07 ^a^	20.82 ± 0.10 ^a^	0.72 ± 0.00 ^b^	3.20
FEP	41.69 ± 0.02 ^c^	15.97 ± 0.03 ^a^	12.68 ± 0.02 ^c^	20.39 ± 0.03 ^b^	0.67 ± 0.00 ^d^	2.07

The values are expressed as the mean ± standard deviation. The presence of the same superscript letter (a–d) in each column indicates that there is no statistically significant difference between the values (*p* < 0.05). F0—fruit purée with oat flakes with the addition of freeze-dried potato starch gel (control sample); FSN—fruit purée with oat flakes enriched with *Sambucus nigra* (elderberry) biocomposites; FAM—fruit purée with oat flakes enriched with *Aronia melanocarpa* (chokeberry fruit) biocomposites; FEP—fruit purée with oat flakes enriched with *Echinacea purpurea* (purple coneflower) biocomposites.

**Table 5:** Representative photo of fruit purée with oat flakes.

Sample	F0	FSN	FAM	FEP
Fruit purée with oat flakes	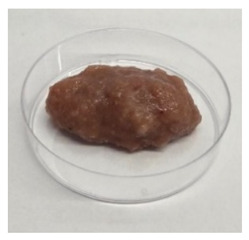	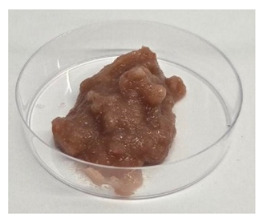	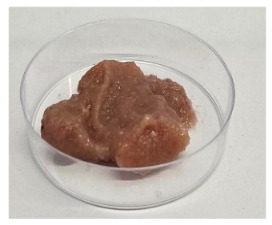	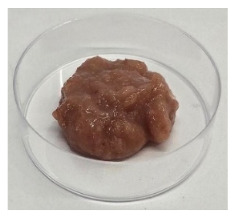

F0—fruit purée with oat flakes with the addition of freeze-dried potato starch gel (control sample); FSN—fruit purée with oat flakes enriched with *Sambucus nigra* (elderberry) biocomposites; FAM—fruit purée with oat flakes enriched with *Aronia melanocarpa* (chokeberry fruit) biocomposites; FEP—fruit purée with oat flakes enriched with *Echinacea purpurea* (purple coneflower) biocomposites.

**Table 6:** The yield point (τ_10%_) obtained from stress sweep tests.

Sample	τ_10%_ (Pa)
F0	37.99
FSN	53.96
FAM	37.89
FEP	40.12

F0—fruit purée with oat flakes with the addition of freeze-dried potato starch gel (control sample); FSN—fruit purée with oat flakes enriched with *Sambucus nigra* (elderberry) biocomposites; FAM—fruit purée with oat flakes enriched with *Aronia melanocarpa* (chokeberry fruit) biocomposites; FEP—fruit purée with oat flakes enriched with *Echinacea purpurea* (purple coneflower) biocomposites.

**Table 7:** Rheological model parameters fitted to flow curves and area of hysteresis loops of fruit purée with oat flakes.

Sample	Ostwald de-Waele Model			Area of the Hysteresis Loop (Pa/s)
	*K* (Pa·s^n^)	*n* (–)	R^2^	
F0	142.91 ± 14.52 ^a^	0.07 ± 0.00 ^a^	0.9451	3329.50 ± 178.93 ^a^
FSN	134.25 ± 12.94 ^a^	0.07 ± 0.01 ^a^	0.9333	2759.50 ± 323.15 ^b^
FAM	149.07 ± 1.65 ^a^	0.05 ± 0.00 ^a^	0.8250	3138.50 ± 163.34 ^ab^
FEP	131.87 ± 10.95 ^a^	0.07 ± 0.02 ^a^	0.9475	2896.00 ± 255.51 ^b^

The values are expressed as the mean ± standard deviation. The presence of the same superscript letter (a, b) in each column indicates that there is no statistically significant difference between the values (*p* < 0.05). F0—fruit purée with oat flakes with the addition of freeze-dried potato starch gel (control sample); FSN—fruit purée with oat flakes enriched with *Sambucus nigra* (elderberry) biocomposites; FAM—fruit purée with oat flakes enriched with *Aronia melanocarpa* (chokeberry fruit) biocomposites; FEP—fruit purée with oat flakes enriched with *Echinacea purpurea* (purple coneflower) biocomposites.

**Table 8:** Textural parameters of fresh fruit purée with oat flakes.

Sample	Hardness (N)	Adhesiveness (N·s)	Springiness (-)	Cohesiveness (-)	Gumminess (-)
F0	1.17 ± 0.08 ^a^	8.20 ± 0.70 ^a^	0.91 ± 0.07 ^a^	0.61 ± 0.04 ^b^	0.71 ± 0.06 ^b^
FSN	1.23 ± 0.14 ^a^	8.17 ± 0.36 ^a^	0.96 ± 0.00 ^a^	0.62 ± 0.04 ^b^	0.84 ± 0.07 ^a^
FAM	1.23 ± 0.12 ^a^	8.95 ± 0.75 ^a^	0.95 ± 0.04 ^a^	0.66 ± 0.06 ^a^	0.85 ± 0.08 ^a^
FEP	1.20 ± 0.06 ^a^	8.37 ± 0.80 ^a^	0.93 ± 0.04 ^a^	0.70 ± 0.01 ^a^	0.83 ± 0.04 ^a^

The values are expressed as the mean ± standard deviation. The presence of the same superscript letter (a, b) in each column indicates that there is no statistically significant difference between the values (*p* < 0.05). F0—fruit purée with oat flakes with the addition of freeze-dried potato starch gel (control sample); FSN—fruit purée with oat flakes enriched with *Sambucus nigra* (elderberry) biocomposites; FAM—fruit purée with oat flakes enriched with *Aronia melanocarpa* (chokeberry fruit) biocomposites; FEP—fruit purée with oat flakes enriched with *Echinacea purpurea* (purple coneflower) biocomposites.

**Table 9:** Percentage of strains the growth of which was inhibited by the application of different purée types with distinction of Gram-positive and Gram-negative groups.

Gram Type	Product Type			
	FAM	FSN	FEP	F0
Gram-positive (n = 33)	18.2	21.2	36.4	3.0
Gram-negative (n = 25)	32.0	36.0	44.0	4.0
Total (n = 58)	24.1	27.6	39.7	3.4

**Table 10:** The content of volatile compounds in the analyzed samples determined using the headspace method on E-nose (HERACLES2/Neo). The concentrations were expressed as percentages relative to the volatile fraction above the analyzed sample.

Volatile Compound	F0	FSN	FAM	FEP	Literature Reference
	Aldehydes [%]				
Acetaldehyde	2.01 ^b^ ± 0.11	2.21 ^c^ ± 0.01	2.23 ^c^ ± 0.09	1.84 ^a^ ± 0.03	[84,85,86]
Hexanal	15.13 ^b^ ± 0.35	15.29 ^b,c^ ± 0.09	15.87 ^c^ ± 0.21	12.93 ^a^ ± 0.23	[87,88,89,90,91,92,93]
Methional	0.08 ^b^ ± 0.00	0.07 ^a^ ± 0.00	—	—	[94]
Sum	17.22	17.57	18.10	14.77	
	Alcohols [%]				
Ethanol	11.61 ^b^ ± 0.69	13.30 ^c^ ± 0.53	13.80 ^c^ ± 0.32	8.73 ^a^ ± 0.25	[85,86,91]
2-Methyl-1-propanol	1.00 ^b^ ± 0.08	0.87 ^a^ ± 0.02	0.96 ^b^ ± 0.03	1.03 ^b^ ± 0.05	[86,95]
2-Methyl-1-butanol	—	—	1.12 ± 0.02	—	[84,96]
Heptan-2-ol	0.18 ^a^ ± 0.01	0.13 ^a^ ± 0.04	0.17 ^a^ ± 0.02	0.82 ^b^ ± 0.07	[86,91,97]
Linalool	—	—	0.16 ^b^ ± 0.02	0.08 ^a^ ± 0.00	[87,93,98,99]
Sum	12.79	14.30	16.21	10.66	
	Ketones [%]				
Propan-2-on	1.92 ^b^ ± 0.14	1.50 ^a^ ± 0.18	1.53 ^a^ ± 0.03	1.71 ^a,b^ ± 0.06	[85]
Sum	1.92	1.50	1.53	1.71	
	Esters [%]				
Buthyl acetate	9.16 ^a^ ± 0.12	9.82 ^b^ ± 0.10	9.69 ^b^ ± 0.05	8.91 ^a^ ± 0.19	[85,90,91,93,100]
Ethyl acetate	12.97 ^b^ ± 0.30	13.35 ^b^ ± 0.24	16.34 ^c^ ± 0.23	11.39 ^a^ ± 0.24	[84,85,91,92,95,96]
Ethyl hexanote	1.30 ^a^ ± 0.04	1.20 ^a^ ± 0.06	2.31 ^b^ ± 0.08	2.09 ^b^ ± 0.14	[91,92,93,98,99,101]
Ethyl heptanoate	0.18 ^a^ ± 0.02	0.22 ^a^ ± 0.02	—	0.43 ^b^ ± 0.04	n.d.
Methy 2-methylbutanoate	2.08 ^b^ ± 0.09	2.13 ^b^ ± 0.07	0.92 ^a^ ± 0.06	3.40 ^c^ ± 0.08	[84,85,89,96]
Ethyl 2-methylbutanoate	1.62 ^c^ ± 0.04	1.42 ^b^ ± 0.05	1.11 ^a^ ± 0.01	1.88 ^d^ ± 0.11	[90,95]
Ethyl octanoate	0.17 ^b^ ± 0.00	0.14 ^a^ ± 0.01	0.20 ^b^ ± 0.00	0.27 ^c^ ± 0.01	[90,98,100]
Hexyl acetate	3.55 ^a^ ± 0.09	4.69 ^b^ ± 0.30	3.41 ^a^ ± 0.16	3.47 ^a^ ± 0.27	[85,86,89,90,93,96]
Isoamyl acetate	8.96 ^b^ ± 0.21	9.12 ^b^ ± 0.43	4.58 ^a^ ± 0.21	10.77 ^c^ ± 0.40	[86,90,95,97,100]
Pentyl octanoate	0.05 ^a^ ± 0.05	0.05 ^a^ ± 0.00	0.06 ^a^ ± 0.05	0.06 ^a^ ± 0.00	n.d.
Sum	40.04	42.41	38.62	42.70	
	Organic acids [%]				
Pentanoic acid	—	—	0.35 ± 0.02	—	[95]
Sum	0.0	0.0	0.35	0.0	
	Terpenoids [%]				
β-Damascenone	—	—	—	0.03 ± 0.00	n.d.
α-Myrcene	—	—	—	0.08 ± 0.01	[102,103]
α-Terpineol	—	0.09 ^a^ ± 0.01	—	0.15 ^b^ ± 0.01	[99,104]
Sum	0.0	0.09	0.20	0.26	

The values are expressed as the mean ± standard deviation. The presence of the same superscript letter (a–d) in each row indicates that there is no statistically significant difference between the values (*p* < 0.05). n.d.—Not detected.

**Table 11:** Retention times of the analyzed volatile compounds on two columns, odor detection threshold values (detection type), and sensory descriptor characteristics.

Volatile Compound	Retention Time MXT-5 [Min]	Retention Time MXT-1701 [Min]	Odor * Threshold [mg/m^3^]	Sensory Descriptors *
	Aldehydes			
Acetaldehyde	14.71	17.90	0.08	Apple; Floral; Fresh; Fruity
Hexanal	40.39	51.37	0.005	Fruity; Grassy; Herbaceous; Leafy; Vinous
Methional	51.63	63.48	0.0004	Creamy; Earthy; Grassy; Musty; Vegetable
	Alcohols			
Ethanol	16.72	21.28	154	Alcoholic; Etheral; Pleasant; Pungent; Sweet
2-Methyl-1-propanol	23.08	35.13	2.32	Alcoholic; Chemical; Licorice; Sweet; Winey
2-Methyl-1-butanol	33.44	46.20	0.14	Alcoholic; Balsamic; Banana; Malty; Sweet; Vinous
Heptan-2-ol	50.92	62.10	0.10	Acrid; Cheese
Linalool	67.29	74.01	0.002	Citrus; Floral; Fruity; Spicy; Sweet; Terpenic;
	Ketones			
Propan-2-on	17.64	22.62	48	Apple; Fruity; Pear; Solvent; Sweet
	Esters			
Butyl acetate	41.93	51.42	0.7	Banana; Etheral; Fruity; Strong; Sweet
Ethyl acetate	21.91	29.26	5.08	Caramelized; Etheral; Fruity; Pineapple; Sweet
Ethyl hexanoate	59.29	65.16	0.03	Anise; Banana; Berry; Fruity; Waxy;
Ethyl heptanoate	66.14	72.06	0.19	Fruity
Methyl- 2-methylbutanoate	37.22	46.27	1.18	Apple; Fatty; Fruity; Lily; Powdery; Alcoholic
Ethyl 2-methylbutanoate	45.81	53.20	0.0012	Apple; Fruity; Sharp; Strawberry; Sweet
Ethyl octanoate	72.61	77.53	0.02	Anise; Floral; Fruity; Leafy; Mentholic; Waxy;
Hexyl acetate	60.98	66.75	0.08	Acidic; Fruity; Herbaceous; Rubber; Spicy; Sweet;
Isoamyl acetate	48.64	56.27	0.14	Apple; Banana; Fresh; Fruity; Pear; Sweet
Pentyl octanoate	88.44	92.02	n.d.	Fatty; Orris; Sweet; Winey
	Organic acids			
Pentanoic acid	52.34	68.11	0.01	Acidic; Cheese; Pungent; Putrid; Rancid;
	Terpenoids			
β-Damascenone	83.88	89.12	0.00004	Apple; Fruity; Honey; Rose; Sweet;
Myrcene	59.94	63.41	0.04	Balsamic; Fruity; Etheral; Musty; Lemon; Geranium
α-Terpineol	73.25	79.58	0.19	Anise; Citrus; Floral; Fruity; Minty; Peach; Pine;

n.d.—no data, * the values of odor threshold and characteristics of sensory descriptors were based on AroChemBase v7 (Alpha MOS, Toulouse, France).

**Table 12:** Results of the consumer preference ranking test.

Sample	F0	FSN	FAM	FEP
Rank sums	254 ^a^	272 ^a^	280 ^a^	254 ^a^

Values sharing the same superscript letter do not differ significantly (*p* > 0.05).

**Table 13:** Formulation of the prepared fruit purée with oat flakes.

Ingredients (g)	Control	Product Enriched with Encapsulated Bioactive Substances
	F0	FSN, FAM, FEP
apple	600	600
strawberry	200	200
banana	150	150
oat flakes	50	50
freeze-dried potato starch gel	1	0
biocomposites	0	1

## Data Availability

The data presented in this study are available on request from the corresponding author. The data are not publicly available due to privacy restrictions and ongoing research utilizing the same dataset.

## Supplementary Materials

The following supporting information can be downloaded at: https://www.mdpi.com/article/10.3390/ijms27135685/s1.

## Abbreviations

The following abbreviations are used in this manuscript:

SEM	Scanning Electron Microscopy
FTIR	Fourier-Transform Infrared Spectroscopy
CIE Lab*	Commission Internationale de l’Éclairage Lightness and Color Coordinates
TPC	Total Polyphenol Content
TFC	Total Flavonoid Content
HPLC	High-Performance Liquid Chromatography
AA	Antioxidant Activity
GAE	Gallic Acid Equivalents
QE	Quercetin Equivalents
TE	Trolox Equivalents
FRAP	Ferric ion Reducing Antioxidant Parameter
ABTS	2,2′-azinobis(3-ethylbenzothiazoline-6-sulfonic acid) radical cation decolorization assay
DPPH	2,2-diphenyl-1-picrylhydrazyl radical scavenging assay
