# Supplementary material for: Design of Functional Food Containing Encapsulated Bioactive Compounds Stabilized in a Psyllium–Potato Starch System
Source: Int J Mol Sci. 2026 Jun 24;27(13):5685. doi: 10.3390/ijms27135685 (PMC13360890; doi:10.3390/ijms27135685)
Supplement: Supplementary file 1 [file ijms-27-05685-s001.zip › ijms-4365602-supplementary.pdf]

Table S1. Results of growth inhibition.

Gram	Identification	FAM	FSN	FEP	F0
G+	Bacillus subtilis	0	0	0	0
G+	Enterococcus faecium	0	0	0	0
G+	Staphylococcus haemolyticus	0	0	0	0
G+	Staphylococcus epidermidis	0	0	0	0
G+	Staphylococcus epidermidis	0	0	0	0
G+	Staphylococcus epidermidis	0	0	0	0
G+	Staphylococcus saprophyticus	0	0	16	0
G+	Staphylococcus warneri	12	10	11	0
G+	Staphylococcus warneri	0	10	9	0
G+	Staphylococcus warneri	0	0	0	0
G+	Staphylococcus warneri	8	0	8	0
G+	Staphylococcus aureus	0	0	14	0
G+	Staphylococcus aureus	0	0	14	0
G+	Staphylococcus aureus	0	0	0	0
G+	Staphylococcus aureus	0	0	0	0
G+	Staphylococcus aureus	11	0	0	0
G+	Staphylococcus aureus	0	0	0	0
G+	Staphylococcus aureus	0	0	0	0
G+	Staphylococcus aureus	0	0	0	0
G+	Staphylococcus aureus	0	0	0	0
G+	Staphylococcus aureus	0	0	0	0
G+	Staphylococcus aureus	0	11	10	0
G+	Staphylococcus aureus	9	9	13	9
G+	Streptococcus mitis	0	22	21	0
G+	Streptococcus mitis	0	0	0	0
G+	Streptococcus salivarius	0	0	14	0
G+	Streptococcus pneumoniae	0	0	0	0
G+	Streptococcus pneumoniae	0	0	0	0
G+	Streptococcus pneumoniae	0	14	0	0
G+	Streptococcus C-group	11	0	0	0
G+	Streptococcus dysgalactiae	0	0	13	0
G+	Streptococcus dysgalactiae	0	0	0	0
G+	Streptococcus pyogenes	8	9	11	0
G-	Neisseria flavescens	0	12	12	0
G-	Moraxella catarrhalis	10	0	16	0
G-	Moraxella catarrhalis	0	0	22	0
G-	Klebsiella pneumoniae	0	0	0	0

G-	Klebsiella oxytoca	0	0	0	0
G-	Klebsiella pneumoniae	12	0	0	0
G-	Proteus mirabilis	11	0	0	0
G-	Escherichia coli	0	0	0	0
G-	Escherichia coli	0	0	7	0
G-	Escherichia coli	0	0	0	0
G-	Haemophilus influenzae	0	0	0	0
G-	Acinetobacter pittii	8	18	22	0
G-	Pseudomonas aeruginosa	0	0	0	0
G-	Pseudomonas rhodesiae	7	9	7	0
G-	Pseudomonas synthaxa	0	7	0	0
G-	Pseudomonas koreensis	27	23	29	21
G-	Pantoea agglomerans	0	0	0	0
G-	Aeromonas veronii	0	9	9	0
G-	Aeromonas salmonicida	0	16	8	0
G-	Aeromonas eucrenophila	0	0	0	0
G-	Aeromonas eucrenophila	0	0	0	0
G-	Aeromonas eucrenophila	7	7	7	0
G-	Aeromonas bestiarum	0	0	0	0
G-	Aeromonas bestiarum	0	0	0	0
G-	Aeromonas bestiarum	7	13	13	0

F0 - fruit purée with oat flakes, with the addition of freeze-dried potato starch gel (control sample).
FSN - fruit mousse enriched with Sambucus nigra (elderberry) biocomposites.
FAM - fruit purée with oat flakes e enriched with Aronia melanocarpa (chokeberry fruit) biocomposites.
FEP - fruit purée with oat flakes enriched with Echinacea purpurea (purple coneflower) biocomposites.
